# Effects of homozygous PARK7 gene mutations L166P and M26I on BNIP3/BNIP3L interactions and ER-mitochondria proximity

**DOI:** 10.1186/s13062-026-00928-8

**Published:** 2026-08-05

**Authors:** Jiannan Wu, Kuiqi Jin, Mengmeng Shen, Xiaoyi Lai, Jingjing Guo, Tingting Wang, Zhenzhen Tian, Junqiang Yan

**Affiliations:** 1 Key Laboratory of Neuromolecular Biology of Henan Province, The First Affiliated Hospital, and College of Clinical Medicine of Henan University of Science and Technology, Luoyang, 471003 China; 2 Key Laboratory of Neural Regeneration of Luoyang, The First Affiliated Hospital, and College of Clinical Medicine of Henan University of Science and Technology, Luoyang, 471003 China; 3 Henan Key Laboratory of Synthetic Biology and Biomanufacturing, School of Life Sciences, Henan University, Kaifeng, 475004 China; 4 Engineering Research Center for Applied Microbiology of Henan Province, School of Life Sciences, Henan University, Kaifeng, 475004 China; 5 Henan Key Laboratory of Cardiovascular Science, Department of Cardiology, The First Affiliated Hospital of Henan University of Science and Technology, Luoyang, 471003 China; 6 Department of Neurology, The First Affiliated Hospital, College of Clinical Medicine of Henan, University of Science and Technology, Luoyang, 471003 China

**Keywords:** PARK7, DJ-1, Mutant, Mitochondria, Endoplasmic reticulum, BNIP3, BNIP3L, Parkinson's disease

## Abstract

Mutations in PARK7, which encodes DJ-1, cause autosomal recessive early-onset Parkinson’s disease. DJ-1 contributes to mitochondrial homeostasis and ER-mitochondria communication, but how the pathogenic L166P and M26I variants affect BNIP3/BNIP3L-associated phenotypes remains incompletely understood. Here, we examined DJ-1 variant-dependent changes in BNIP3/BNIP3L interactions, mitochondrial morphology, ER-mitochondria proximity-associated readouts, and global intracellular Ca^2+^ responses in PC-12 and SH-SY5Y cell models. AlphaFold3-based interface prediction, co-immunoprecipitation, and GST pull-down assays supported interactions involving DJ-1, BNIP3, and BNIP3L, including binding to selected BNIP3- and BNIP3L-derived peptide regions. DJ-1 WT and M26I showed detectable binding to BNIP3-derived regions, whereas the unstable L166P variant showed reduced BNIP3 binding even after partial restoration of L166P abundance with MG132. Reciprocal co-immunoprecipitation and knockdown experiments further supported an association between BNIP3 and BNIP3L in these cells. In L166P expressing cells with GRP75 knockdown, BNIP3 depletion increased the ER-mitochondria distance and reduced the length of the ER-mitochondria proximity region. Colocalization analyses showed DJ-1 variant-dependent changes in proximity-associated imaging readouts, while immunoblotting identified reduced VDAC1 and MFN1 levels after BNIP3 depletion in mutant expressing cells. BNIP3 depletion reduced the relative 2-APB evoked Fluo-4 response in WT, L166P and M26I expressing cells by 40.7%, 76.7%, and 42.5%, respectively. Overall, these findings may reflect altered DJ-1/BNIP3/BNIP3L interaction profiles and BNIP3 sensitive changes in ER-mitochondria proximity associated and global intracellular Ca^2+^ readouts, particularly in L166P-expressing cells, although direct validation is still required.

**Supplementary Information:**

The online version contains supplementary material available at https://doi.org/10.1186/s13062-026-00928-8.

## Introduction

The *PARK7* gene encodes the Parkinson disease protein 7 (Park7), also known as DJ-1. Mutations in this protein are closely associated with the pathogenesis of autosomal recessive early-onset Parkinson’s disease (PD). In healthy individuals, DJ-1 acts as an antioxidant and oxidative stress sensor [1], playing critical roles in various neuroprotective mechanisms. It also participates in maintaining mitochondrial homeostasis, apoptosis regulation, chaperone-mediated autophagy (CMA), and dopamine homeostasis by modulating diverse signaling pathways, transcription factors, and molecular chaperone functions [2]. Its role in regulating mitochondrial function within the context of PD pathogenesis has garnered significant attention, which may be closely linked to DJ-1’s involvement in the structural relationship among mitochondria, the endoplasmic reticulum (ER), and mitochondria-associated ER membranes (MAMs) [3].

MAMs also referred to as mitochondria-ER contact sites (MERCs), involve the physical connections between the ER and mitochondrial membranes. Their functions encompass calcium ion transport, mitochondrial structural regulation, mitophagy, apoptosis, and lipid transfer between membranes—all of which are implicated in the molecular mechanisms of PD pathogenesis. Wild-type DJ-1 forms relatively stable dimers, whereas the L166P mutant exists as a monomer that is rapidly degraded and inactivated via the ubiquitin proteasome pathway. DJ-1 interacts with the canonical MAMs structural complex IP3R3-Grp75-VDAC1; mutations or depletion of DJ-1 disrupt the stability of this complex, leading to a reduction in ER-mitochondria tethering structures [4]. Consequently, this decreases the structural stability of both mitochondria and the ER, and impairs Ca^2+^ transfer from the ER to mitochondria [5]. Although DJ-1 has been shown to be associated with the stabilization of the IP3R3-Grp75-VDAC1 complex, it lacks a specific MAM-targeting sequence. Therefore, how it precisely anchors to MAM structures and whether it possesses interactive functions beyond stabilization remain inconclusive. Furthermore, under conditions of complex instability caused by DJ-1 mutations, the dynamic mechanisms affecting MAM binding sites, affinity, and variations across different physiological states and calcium metabolism remain incompletely understood. In particular, the underlying basis by which mutants like L166P weaken the complex structure requires further investigation.

DJ-1 has previously been linked to ER-mitochondria communication through regulation of the canonical IP3R3-GRP75-VDAC1 axis and related contact site biology. Prior work has also reported DJ-1 dependent modulation of ER-mitochondria contact sites and mitochondrial phenotypes through mechanisms that may not be explained solely by GRP75 dependent regulation [6].Given the close association of DJ-1 and its mutants with mitochondria and the ER, further exploring their alterations in related disease models is of paramount importance. In our previous study on mitophagy induced by quinone compounds in a PD mouse model, we observed an abnormal reduction in mitochondria associated VDAC1 and BNIP3 [7]. BNIP3 (Bcl-2/adenovirus E1B 19-kDa interacting protein 3) is a BH3-only protein of the Bcl-2 family that participates in regulating apoptosis. BNIP3 can interact with both the ER and mitochondria, influencing apoptosis by regulating Ca^2+^ levels between these organelles [8–10]. The dual subcellular localization of BNIP3 at both mitochondria and the ER initiates distinct cell death signaling events, suggesting that BNIP3 likely forms a physical or functional link between the ER and mitochondria. Concurrently, since DJ-1 is positively correlated with the stability of MAM structures involving VDAC1, the key unresolved question addressed here is whether pathogenic PARK7 L166P and M26I variants alter additional protein interaction networks that are associated with ER-mitochondria proximity when canonical MAM related regulation is perturbed.

BNIP3L, a homolog of BNIP3, is localized to the nuclear envelope, ER, and mitochondrial membranes [11]. BNIP3 is localized to the outer ER membrane [12] and the outer mitochondrial membrane (OMM) [13, 14]. Both proteins exhibit significant binding to RIPK3 (Receptor-interacting protein kinase 3) and FBXL4, respectively, to maintain mitochondrial homeostasis [15, 16]. These studies suggest that BNIP3 and BNIP3L are involved in the physical connection between mitochondria and the ER. They establish functional links between the ER and mitochondria either by regulating Ca^2+^ levels which increased BNIP3 expression leads to decreased ER Ca^2+^ and increased mitochondrial Ca^2+^ levels [17], or by participating in autophagy, as BNIP3L mediates mitophagy through its interaction with mitochondria. This mechanism is similar to another MAMs assembly the mitochondrial fusion protein complex (MFN) MFN1/MFN2, where ER resident MFN2 assembles with OMM resident MFN1/2 to form homo- or heterodimeric complexes [18]. However, determining whether they exist as stable physical tethers requires more detailed studies to elucidate the evidence of their interactions between the ER and mitochondria, as well as the specific molecular mechanisms or structural domains involved.

In this study, we investigated the pathogenic PARK7 variants L166P and M26I to determine whether they alter DJ-1 interactions with rat- and human-derived BNIP3 and BNIP3L and whether these alterations are associated with mitochondrial morphology, ER-mitochondria proximity related phenotypes, and global intracellular Ca^2+^ responses. We combined AlphaFold3 based structural prediction with co-immunoprecipitation, GST pull-down assays, fluorescence imaging, transmission electron microscopy, and Fluo-4 measurements to characterize variant dependent protein interaction and cellular phenotypes. Because conventional fluorescence microscopy and transmission electron microscopy provide complementary but indirect or static information on organelle proximity, the corresponding imaging measurements were interpreted as proximity associated readouts. Accordingly, this study aimed to examine how L166P and M26I influence the DJ-1/BNIP3/BNIP3L interaction network and related cellular phenotypes, thereby providing further insight into mitochondrial dysfunction associated with early-onset Parkinson’s disease.

## Materials and methods

### Cell culture

SH-SY5Y and PC-12 cell lines were kindly provided by the Stem Cell Bank, Chinese Academy of Sciences. SH-SY5Y cells were cultured in DMEM/F-12 (Dulbecco’s Modified Eagle Medium/Nutrient Mixture F-12) 50/50 mix (Corning, 10-092-CVRC) supplemented with L-glutamine, 15 mM HEPES, 10% fetal bovine serum (FBS) (Gibco, A31608-02), and 1% non-essential amino acids (Gibco, 11140050). Highly differentiated PC-12 cells were cultured in RPMI 1640 medium (Corning, 10-040-CV) containing L-glutamine and 10% FBS. All cells were maintained in a humidified incubator at 37 °C with 5% CO₂.

### Plasmids and transfection

Plasmids were synthesized and constructed by GenScript (Nanjing, China). The human wild type PARK7 gene (Gene ID: 11315) was cloned into pcDNA3.1-Myc-His and pcDNA3.1-GFP vectors. The human DJ-1 missense mutations (L166P and M26I) were introduced via site directed mutagenesis. Transient transfections were performed using Lipofectamine 3000 (Thermo Fisher, L3000015) according to the manufacturer’s instructions. To establish stable monoclonal cell lines, transfected cells were selected using a culture medium containing G418 (Med Chem Express, HY-17561). Cells expressing GFP tags were observed and imaged using an inverted fluorescence microscope (Nikon, ECLIPSE Ti-E).

### RNA interference (RNAi)

To knock down human GRP75, three short hairpin RNA (shRNA) sequences were specifically designed and cloned into the pLKO.1-puro vector. The target sequences were: sh-GRP75-1, 5’-CGGCGATATGATGATCCTGAA-3’; sh-GRP75-2, 5’-GCTGTCACCAACCCAAACAAT-3’; and sh-GRP75-3, 5’-CGTGCTCAATTTGAAGGGATT-3’. These plasmids, alongside a non-targeting scrambled shRNA (negative control, sh-NC), were transfected into SH-SY5Y cells using Lipofectamine 3000. At 24 h post-transfection, the medium was replaced with a fresh medium containing puromycin (2 µg/mL) to select for stable transfectants. Knockdown efficiency was validated by Western blotting (WB) to establish the stable shRNA-expressing cell lines.

For DJ-1, BNIP3 and BNIP3L knockdown, three pairs of small interfering RNAs (siRNAs) and a negative control siRNA (NC-siRNA) were transfected into SH-SY5Y cells using Lipofectamine 3000. The specific sequences were shown in supplementary Table 1. Cells were cultured for an additional 48 h post-transfection, and the interference efficiency was assessed by WB.

### Western blotting

Cells were lysed in RIPA buffer (Solarbio, R0010) supplemented with 1% PMSF. The lysates were centrifuged at 12,000 × g for 15 min at 4 °C, and the supernatants were collected. Protein concentrations were determined using a BCA Protein Assay Kit (Thermo Fisher, #23228). Equal amounts of total protein (20 µg) were separated by 10% or 12.5% SDS-PAGE (Epizyme, PG112 & PG113) using standard electrophoresis buffer (Servicebio, G2152) and transferred onto 0.22 μm PVDF membranes (Merck Millipore, ISEQ00010) in transfer buffer (Servicebio, G2148). The membranes were blocked with 5% milk (Solarbio, D8340) in TBST (Solarbio, T1085) and incubated overnight at 4 °C with the following primary antibodies diluted in 1% milk/TBST shown in supplementary Table 2. After washing with TBST, the membranes were incubated with HRP-conjugated goat anti-rabbit (Proteintech, RGAR001) or goat anti-mouse (Proteintech, RGAM001) secondary antibodies for 2 h at 37 °C. Protein bands were visualized using an Ultrasensitive Enhanced Chemiluminescence Detection Kit (Proteintech, PK10003) and captured on a Tanon 5200 Chemiluminescent Imaging System. Densitometric analysis was performed using ImageJ (version 1.54p), and statistical analyses were conducted using GraphPad Prism 9.5.0.

### Immunofluorescence

Stably transfected cells were seeded at a density of 2 × 10⁴ cells/well onto glass coverslips (WHB, WHB-24-CS) in 24-well plates (Corning, #3524). Cells were fixed with a cell fixation solution (Solarbio, P1110) and subsequently permeabilized and blocked with PBS containing 10% goat serum (Solarbio, SL038) and 0.25% Triton X-100 (Solarbio, IT9100). Cells were then incubated overnight at 4 °C with the primary antibodies diluted in 1% BSA/PBS (Solarbio, A8010). The primary antibodies used were shown in supplementary Table 3. Following PBS washes, cells were incubated with the corresponding fluorescent secondary antibodies for 2 h at 37 °C: Alexa Fluor 488 goat anti-rabbit (Thermo Fisher, A-11008), Alexa Fluor 555 goat anti-mouse (Thermo Fisher, A-21422), Alexa Fluor 488 goat anti-mouse (Thermo Fisher, A-11001), and Alexa Fluor 555 goat anti-rabbit (Thermo Fisher, A-21428). Nuclei were counterstained with DAPI (Beyotime, C1006) for 5 min. The coverslips were washed with PBS and mounted using an antifading mounting medium (Solarbio, S2100). Images were acquired using a fluorescence microscope (Olympus, BM53).

### Co-immunoprecipitation (Co-IP)

Co-IP assays were performed using an Immunoprecipitation Kit with Protein A + G Magnetic Beads (Beyotime, P2179S). Stably expressing SH-SY5Y cells were lysed, and the lysates were processed using a magnetic rack (DynaMag-2, 12321D) according to the manufacturer’s instructions. The lysates were incubated for 12 h at 4 °C with the following antibodies: rabbit anti-PARK7/DJ-1 (2.0 µg, Proteintech, 11681-1-AP); rabbit anti-BNIP3 (1:100, Cell Signaling Technology, #44060); rabbit anti-BNIP3L (2.0 µg, Abcam, ab264312); rabbit anti-MYC tag (2.0 µg, Proteintech, 16286-1-AP); and rabbit IgG control (2.0 µg, Proteintech, 30000-0-AP). The immunoprecipitated complexes were subsequently eluted and analyzed by SDS-PAGE and immunoblotting.

### GST pull-down assay

The GST-fused peptide construct (cloned into the pGEX-4T-1 vector) was transformed into E. coli BL21 (Cwbio, CW0809S). Transformants were selected using 100 µg/mL ampicillin (Solarbio, A1170), and protein expression was induced with 0.5 mM IPTG (Solarbio, II0130). The bacterial culture was centrifuged at 4,000 × g for 10 min, and the pellet was resuspended and subjected to ultrasonic lysis on ice. The GST pull-down assay was performed using a GST Protein Interaction Pull-Down Kit (Thermo Fisher, #21516) according to the manufacturer’s instructions. The bacterial lysate (bait) was incubated with Glutathione Agarose beads for affinity purification. Lysates from stably overexpressing SH-SY5Y cells (prey) were then added to the purified agaroses and incubated with gentle rocking on a rotating platform for 6 h at 4 °C. The bound proteins were eluted and analyzed by SDS-PAGE and immunoblotting.

### Fluo-4 fluorescence assay

Intracellular Ca^2+^ related fluorescence responses were assessed using a Fluo-4 Calcium Assay Kit (Beyotime, S1061S). Cells were seeded in black 96-well plates at a density of 5,000 cells/well and subjected to the designated transfection treatments. After 24 h, the cells were processed according to the kit’s instructions. Prior to the measurement, cells were stimulated with 10 µM 2-APB (Med Chem Express, HY-W009724). In brief, using the first scan as the baseline, it is labeled as Scan (1) After 1 min, 2-APB is added and a scan is conducted, labeled as Scan (2) Subsequently, scans are performed every minute until 15 scans are completed (For each scan was scanned three times and averaged). Signals were recorded using a BioTek Synergy H1 microplate reader (Agilent) at excitation/emission wavelengths of 490/525 nm and expressed as relative fluorescence units (RFU).

### Transmission electron microscopy (TEM)

Approximately 1 × 10⁷ cells were harvested by centrifugation at 12,000 × g and fixed with 2.5% glutaraldehyde (Solarbio, P1126) followed by post-fixation in 1% osmium tetroxide (Sigma-Aldrich, 251755). The samples were then dehydrated through a graded ethanol series and embedded using an Epoxy Embedding Medium Kit (Sigma-Aldrich, 45359). Ultrathin sections  (60 nm) were cut using an ultramicrotome (Leica, EM UC7), mounted on copper grids, and stained. Images were acquired using a transmission electron microscope (Hitachi, HT7700) operating at 80 kV.

### TEM image analysis

TEM images were analyzed using Fiji ImageJ (version 1.54p) according to a standardized image-analysis protocol [19]. Each image was divided into four quadrants using the Quadrant Picking plugin, and two quadrants were randomly selected for analysis. ER-mitochondria proximity regions were identified in TEM sections as areas in which the ER membrane and the outer mitochondrial membrane were clearly distinguishable and remained closely aligned. Two ultrastructural parameters were quantified. The ER-mitochondria distance was defined as the perpendicular distance between the ER membrane and the outer mitochondrial membrane and was measured by drawing a perpendicular line between the two membranes. The length of the ER-mitochondria proximity region was defined as the length of the continuous segment over which the ER membrane and the outer mitochondrial membrane remained closely aligned and was measured by tracing the segment with the Freehand Line tool. All measurements were recorded using the ROI Manager. Image selection and quantification were performed in a randomized and blinded manner by three independent investigators to minimize selection and measurement bias. For each experimental group, 5 images were analyzed, with 3 replicates per group, and data were presented as 15 individual data points.

### Analysis of protein complex contact interface residues

All standard human protein sequences used in this study, including BNIP3 (Q12983), BNIP3L (O60238), and DJ-1 (Q99497), were downloaded from the UniProt database. Protein complex interactions were predicted using the AlphaFold3 server [20]. The generated protein complex structure files were aligned in PyMOL as described previously [21, 22]. Interface residues were identified and visualized using the InterfaceResidues script (pymolwiki.org/index.php/InterfaceResidues) to annotate potential interactions at the protein-protein interfaces, including contacts, salt bridges, π-π interactions, and van der Waals forces. All structural analyses and visualizations were performed using PyMOL v3.0.3.

### Colocalization and mitochondrial morphology analysis

Colocalization of two proteins within selected regions of interest was calculated using the Colocalization Finder plugin (Version 1.8) and the Coloc 2 plugin in Fiji ImageJ (version 1.54p). Pearson’s correlation coefficient (PCC, r) and Manders’ colocalization coefficients (MCC) were used to quantify spatial overlap. M1 was defined as the fraction of the Calnexin signal overlapping with GRP75, whereas M2 was defined as the fraction of the GRP75 signal overlapping with Calnexin.

Mitochondrial morphology was analyzed using the Mitochondria Analyzer plugin. Raw images were converted to 8-bit format, subjected to background subtraction, and threshold using a weighted mean method (block size = 1.35 μm, C-value = 3). Morphological parameters, including the number of mitochondria, average area, circularity, and network branching, were quantified for individual cells. Three independent experiments were conducted for each condition. In each experiment, 10 cells were analyzed for each condition and the average value was shown in the graphs. In total, 30 cells were analyzed for each condition.

### Data analysis and statistics

Statistical analyses were performed using GraphPad Prism 9.5.0. Data conforming to a normal distribution are presented as the mean +/- standard error of the mean (SEM). Comparisons between two groups were performed using an unpaired Student’s t-test, while comparisons among multiple groups were analyzed using one-way or two-way analysis of variance (ANOVA) followed by Tukey’s post hoc test. A *P*-value < 0.05 was considered statistically significant.

## Results

### Pathogenic DJ-1 mutants exhibit reduced protein stability and alter the expression and oligomerization of BNIP3 and BNIP3L

Previous studies utilizing autophagy reporter mice demonstrated that quinone antioxidants alter the expression of mitochondria-associated VDAC1 and BNIP3 [7]. Given that wild-type (WT) DJ-1 (encoded by *PARK7*) exerts neuroprotective antioxidant functions, whereas the recessive homozygous mutations L166P and M26I are pathogenic loss function variants, we sought to investigate the impact of DJ-1 and its mutants on ER-mitochondrial homeostasis.

**Fig. 1:**
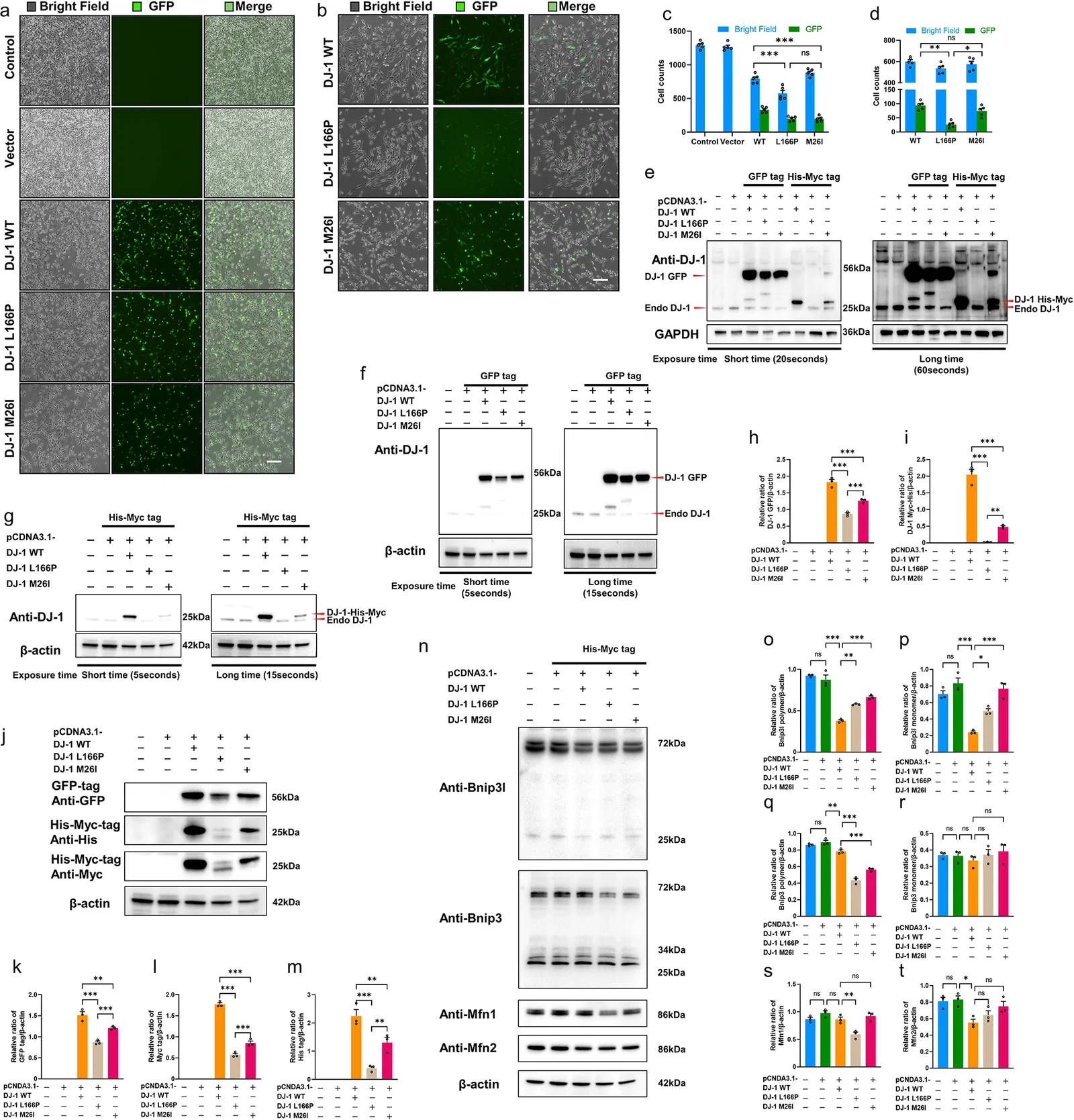
Expression and protein profiles of DJ-1 WT, L166P and M26I in PC-12 cells. PC-12 cells were transfected with pcDNA3.1 plasmids encoding GFP or Myc-His tagged DJ-1 WT, L166P and M26I. **a** Representative bright-field and fluorescence images of PC-12 cells at 48 h after transient transfection. **b** Representative bright-field and fluorescence images of PC-12 cells after stable selection with G418. (Scale bar = 500 μm). **c** Quantification of GFP positive cell counts following transient transfection. **d** Quantification of GFP positive cell counts following stable selection. **e** Western blot (WB) analysis of exogenous DJ-1 expression in transiently transfected PC-12 cells. **f**,** g** Western blot analysis of DJ-1 WT, L166P and M26I expression in PC-12 cells stably expressing GFP tagged DJ-1 (**f**) or Myc-His tagged DJ-1 (**g**). **h**,** i** Quantitative densitometric analysis of the DJ-1 protein expression levels shown in (**f**) and (**g**). **j** WB analysis verifying the expression levels of the GFP tags and Myc-His tags in the stable cell lines. **k-m** Quantitative densitometric analysis of the tagged DJ-1 protein expression levels shown in (**j**). **n** WB analysis of Bnip3l, Bnip3, Mfn1, and Mfn2 expression levels in PC-12 cells stably expressing Myc-His tagged DJ-1 WT, L166P and M26I. **o**-**t** Quantitative densitometric analysis of the protein levels in (**n**). For **c** and **d** data are presented as mean +/- SEM, *N* = 5. *P < 0.05, **P < 0.01, ***P < 0.001; ns,* not significant*. For **h**,** i**,** k**-**m**, and **o-t**, data are presented as mean +/- SEM, *N* = 3. *P < 0.05, **P < 0.01, ***P < 0.001; *ns*,* not significant*

We first overexpressed human DJ-1 (WT, L166P, and M26I) fused with a GFP tag in PC-12 cells. At 48 h post transient transfection, the number of GFP positive cells showed no significant differences between the L166P and M26I group (Fig. 1a, c). However, following G418 selection for one week to establish stable monoclonal cell lines, the proportion of GFP-positive cells in the L166P group was significantly reduced compared to the WT and M26I groups (Fig. 1b, d). To exclude potential artifacts caused by the molecular weight of the GFP tag, we performed parallel transient transfections using Myc-His tagged DJ-1 plasmids. Immunoblotting revealed that while GFP tagged WT, L166P, and M26I, as well as Myc-His tagged WT and M26I, exhibited robust expression under short exposure, the Myc-His tagged L166P mutant displayed only faint signals even under long exposure (Fig. 1e).

Protein expression analysis in the stable PC-12 cells further corroborated this instability. Under a short exposure time (5s), the L166P-GFP protein exhibited distinct degradation patterns compared to WT-GFP and M26I-GFP (Fig. 1f, h). Similarly, WT-His-Myc showed the highest expression level, whereas the levels of M26I and particularly L166P were dramatically decreased; notably, L166P-His-Myc was almost undetectable and showed obvious degradation bands (Fig. 1g, i, j, k, l and m). Overall, the exogenous expression of DJ-1 mutants was notably lower than that of WT, and the Myc-His tagged fusion proteins were generally more susceptible to degradation than their GFP tagged counterparts.

Next, we examined the effects of exogenous DJ-1 on mitochondria associated proteins in these stable cells. Compared to normal PC-12 cells and empty vector controls, the introduction of exogenous DJ-1 decreased the levels of both BNIP3L oligomers (~ 72 kDa) and monomers (~ 25 kDa) (Fig. 1n, o, p). Among the DJ-1 expressing groups, the WT group exhibited the lowest BNIP3L levels, whereas the M26I group retained the highest levels of both oligomers and monomers. Interestingly, the expression pattern of BNIP3 exhibited distinct features: while BNIP3 monomer levels (25–34 kDa) showed no significant alterations across the WT, L166P, and M26I groups, its ~ 72 kDa oligomers were significantly reduced (Fig. 1n, q, r). Furthermore, overexpression of the L166P mutant specifically downregulated the expression of Mfn1, whereas Mfn2 levels remained unaffected across all groups (Fig. 1n, s, t).

### DJ-1 L166P and M26I alter mitochondrial morphology and Bnip3/Bnip3l colocalization in PC-12 cells

**Fig. 2:**
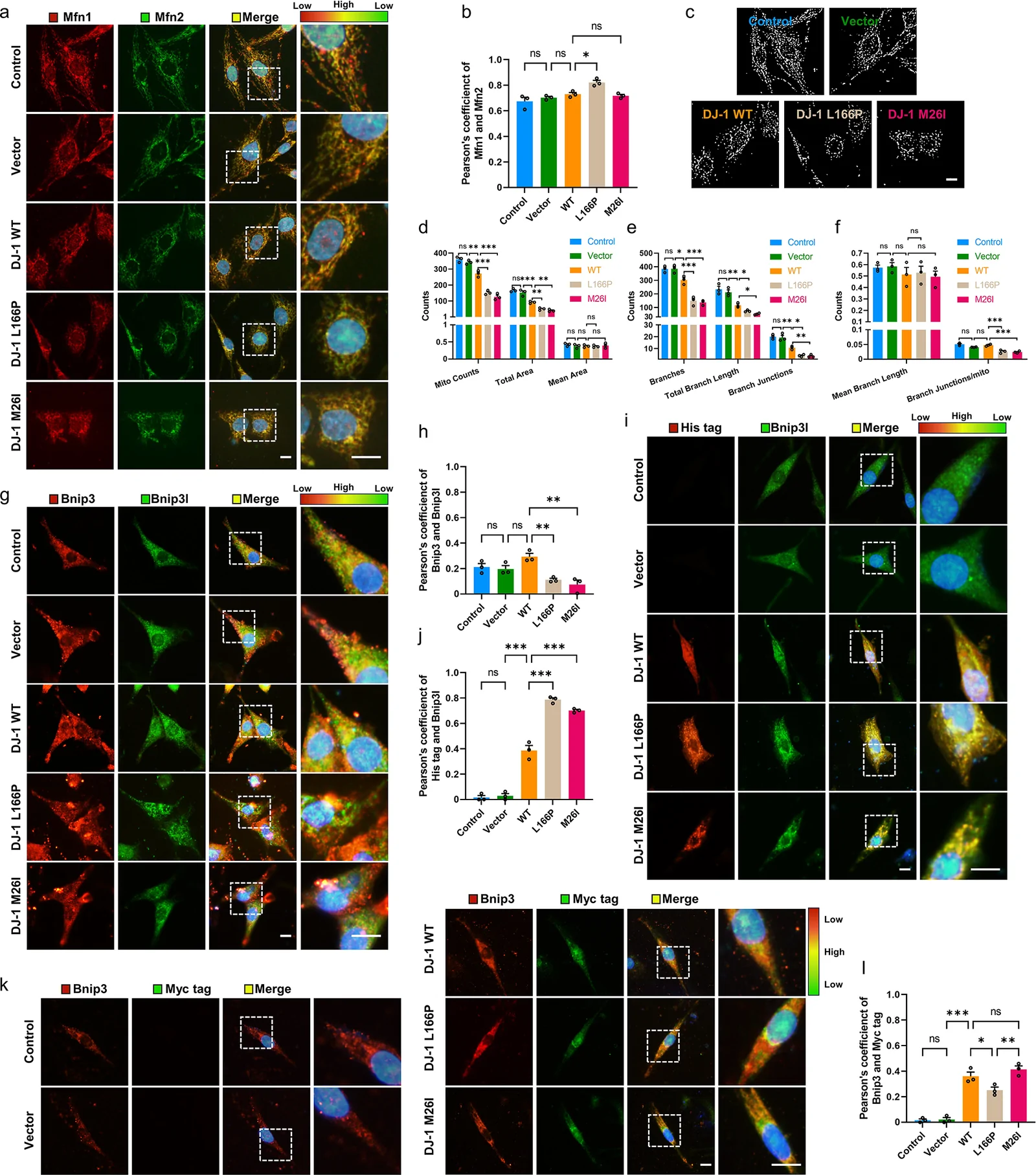
Mitochondrial morphology and protein colocalization in PC-12 cells expressing DJ-1 WT, L166P, or M26I. Assays were performed in PC-12 cells stably expressing Myc-His tagged DJ-1 WT, L166P or M26I. **a** Representative fluorescence images showing Mfn1 (red) and Mfn2 (green). Yellow regions in the merged images indicate overlapping red and green fluorescence signals. **b** Pearson’s correlation coefficient (PCC) analysis of Mfn1 and Mfn2 spatial overlap. **c** Representative images of mitochondrial morphology. **d** Quantification of mitochondrial number, projected total area, and mean mitochondrial area. **e** Quantification of branch number, total branch length, and branch junction number. **f** Quantification of mean branch length and branch junction number per mitochondrion. **g** Representative fluorescence images showing Bnip3(red) and Bnip3l(green). **h** PCC analysis for Bnip3 and Bnip3l colocalization. **i** Representative fluorescence images showing His tagged DJ-1 (red) and Bnip3l (green). **j** PCC analysis for DJ-1 and Bnip3l colocalization. **k** Representative fluorescence images showing Bnip3 (red) and Myc tagged DJ-1 (green). **l** PCC analysis for Bnip3 and DJ-1 colocalization. Pseudocolored heatmaps show the spatial distribution of overlapping fluorescence signals. Scale bars = 10 μm. Data are presented as mean +/- SEM, *N* = 3. *P < 0.05, **P < 0.01, ***P < 0.001; *ns*,* not significant*

MFN1 and MFN2 are mitochondrial fusion proteins that have been implicated in ER-mitochondria organization. Given the reduced stability of the L166P variant, we examined mitochondrial morphology and the intracellular spatial overlap of Mfn1 and Mfn2 in PC-12 cells stably expressing Myc-His-tagged DJ-1 WT, L166P, or M26I. PCC values for Mfn1 and Mfn2 exceeded 0.7 in all groups and 0.8 in the L166P group (Fig. 2a, b), indicating a high degree of signal correlation in the analyzed images.

Mitochondrial morphology analysis and the corresponding quantification revealed differences among the DJ-1-expressing groups (Fig. 2c-f). The mitochondrial number and projected total area were significantly lower in the L166P and M26I expressing groups than in the WT expressing group, whereas the mean area of individual mitochondria did not differ significantly among the groups (Fig. 2d). The number of mitochondrial branches, total branch length, and number of branch junctions were also significantly lower in the L166P and M26I expressing groups than in the WT expressing group (Fig. 2e). Mean branch length did not differ significantly among the groups, whereas the number of branch junctions per mitochondrion was significantly lower in the L166P and M26I expressing groups than in the WT expressing group (Fig. 2f).

We next examined the regional spatial overlap of Bnip3 and Bnip3l signals in cells expressing the different DJ-1 variants. The Bnip3/Bnip3l PCC was significantly higher in DJ-1 WT expressing cells than in normal PC-12 cells and vector control cells (Fig. 2g, h). By contrast, the PCC values in L166P and M26I expressing cells were significantly lower than that in the WT expressing group.

We also quantified the spatial overlap of tagged DJ-1 with Bnip3 and Bnip3l. PCC analysis using the His-tag signal showed significantly higher DJ-1/Bnip3l colocalization in the L166P and M26I expressing groups than in the WT expressing group (Fig. 2i, j). In the DJ-1/Bnip3 analysis using the Myc-tag signal, the L166P expressing group showed a significantly lower PCC than the WT and M26I expressing groups (Fig. 2k, l).

Overall, L166P and M26I expressing cells showed higher DJ-1/Bnip3l PCC values than WT expressing cells, whereas the L166P expressing group showed a lower DJ-1/Bnip3 PCC. These results identify DJ-1 variant dependent differences in the regional spatial overlap of the analyzed signals.

### DJ-1 variants are associated with changes in BNIP3/BNIP3L colocalization and expression in SH-SY5Y cells

To determine whether the colocalization patterns observed in rat PC-12 cells were also present in a human cell model, we examined BNIP3 and BNIP3L (Bnip3 and Bnip3l for rat; BNIP3 and BNIP3L for human) in SH-SY5Y cells expressing DJ-1 WT, L166P, or M26I.

**Fig. 3:**
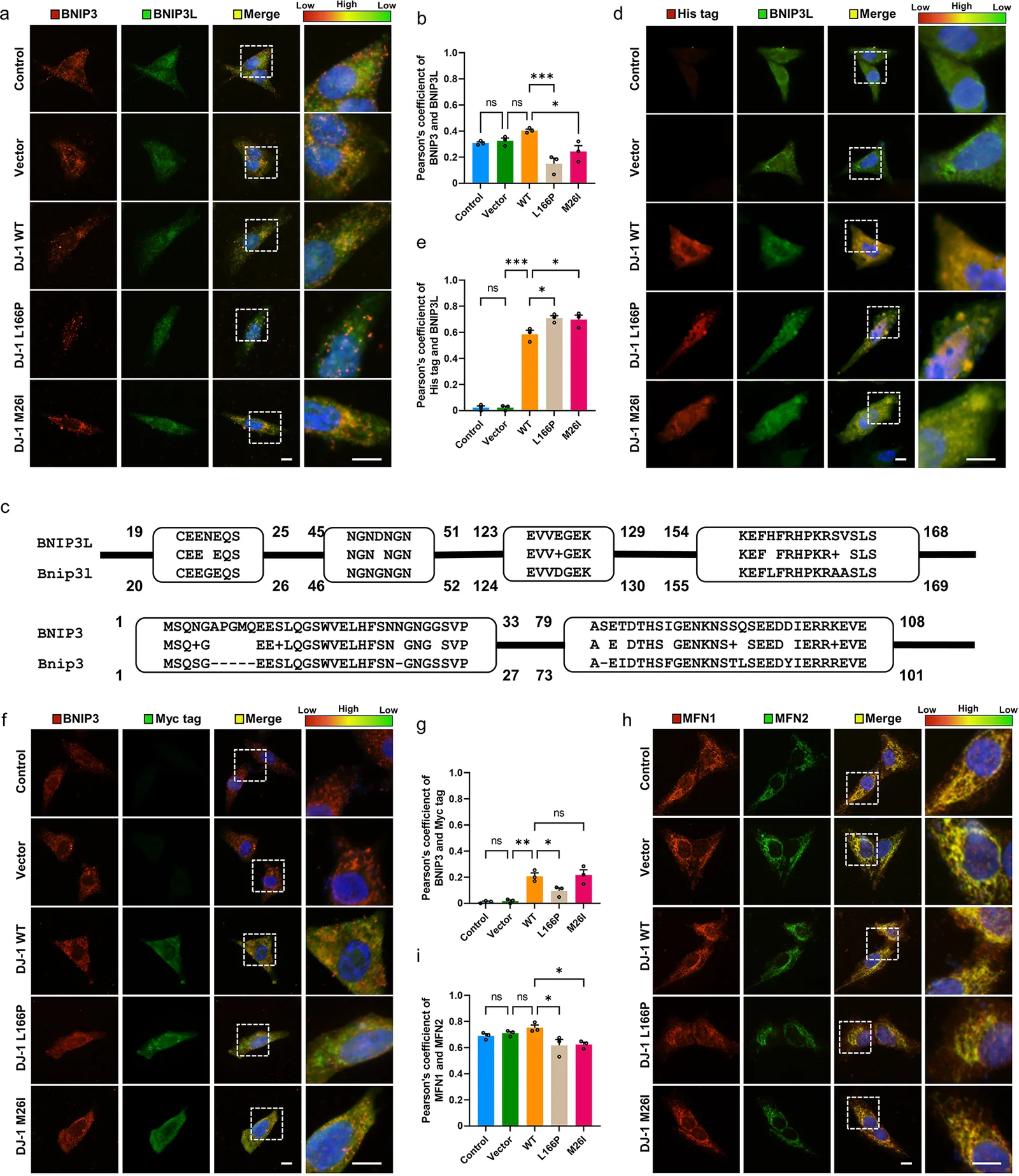

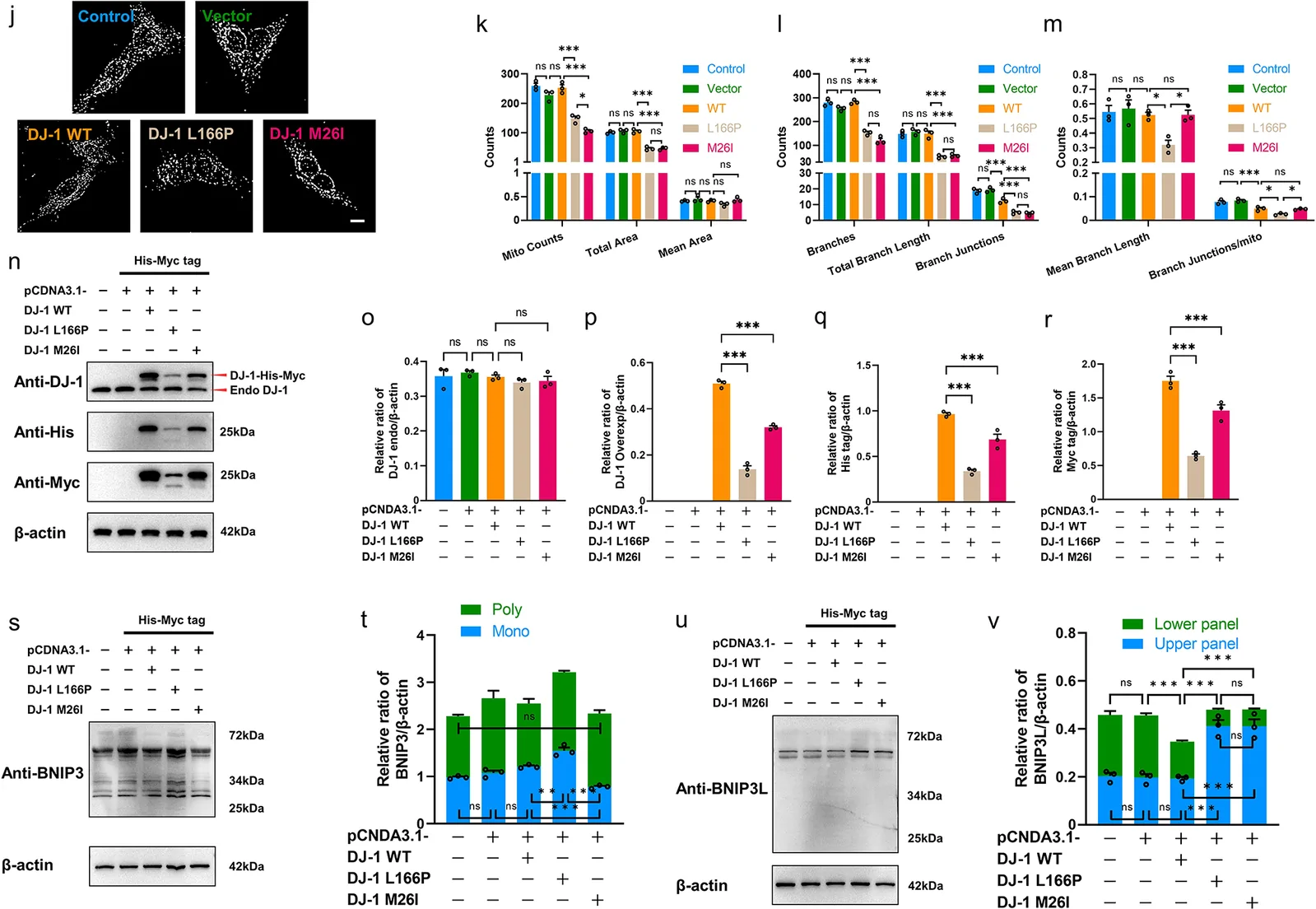
Mitochondrial morphology, protein colocalization, and expression profiles in SH-SY5Y cells expressing DJ-1 variants. SH-SY5Y cells were transfected to stably express His-Myc tagged DJ-1 WT, L166P or M26I. **a** Representative fluorescence images showing BNIP3 (red) and BNIP3L (green). Yellow regions in the merged images indicate overlapping red and green fluorescence signals. **b** PCC analysis for BNIP3 and BNIP3L colocalization. **c** Sequence alignments of human BNIP3 and BNIP3L with their corresponding rat proteins, Bnip3 and Bnip3l. **d** Representative fluorescence images showing His tagged DJ-1 (red) and BNIP3L (green). **e** PCC analysis for DJ-1 and BNIP3L colocalization. **f** Representative fluorescence images showing BNIP3 (red) and Myc-tagged DJ-1 (green). **g** PCC analysis for BNIP3 and DJ-1 colocalization. **h** Representative fluorescence images showing MFN1 (red) and MFN2 (green). **i** PCC analysis for MFN1 and MFN2. **j** Representative images of mitochondrial morphology. **k** Quantification of mitochondrial number, projected total area, and mean mitochondrial area. **l** Quantification of branch number, total branch length, and branch junction number. **m** Quantification of mean branch length and branch junction number per mitochondrion. **n** Western blot analysis confirming the expression of exogenous DJ-1 tags. **o-r** Quantitative analysis of endogenous/exogenous DJ-1 and tag expression levels. **s** Western blot analysis of BNIP3 protein expression. **t** Quantification of BNIP3 oligomers (green bars), monomers (blue bars), and total BNIP3 levels. **u** Western blot analysis of BNIP3L protein expression. **v** Quantification of the lower panel (green bars), upper panel (blue bars), and total BNIP3L levels. Pseudocolored heatmaps show the spatial distribution of overlapping fluorescence signals. Scale bars = 10 μm. Data are presented as mean +/- SEM, *N* = 3. *P < 0.05, **P < 0.01, ***P < 0.001; *ns*,* not significant*)

Consistent with the PC-12 model, the colocalization of BNIP3 and BNIP3L was significantly reduced under DJ-1 L166P overexpression compared to the DJ-1 WT and M26I groups (Fig. 3a, b) in SH-SY5Y cells. Sequence alignment showed high similarity between human and rat BNIP3L, with differences at only six amino acid positions (Fig. 3c). Further analysis in SH-SY5Y cells corroborated that the L166P and M26I mutations significantly increased colocalization with BNIP3L compared to WT DJ-1. (Fig. 3d, e), while the L166P mutant significantly reduced colocalization with BNIP3 compared to the WT and M26I groups (Fig. 3f, g).

Next, we evaluated the impact of exogenous DJ-1 on mitochondria in SH-SY5Y cells. Immunofluorescence and colocalization analysis of MFN1 and MFN2 (Fig. 3h) revealed a significant decrease in PCC following L166P and M26I treatment compared to WT DJ-1 (Fig. 3i). Mitochondrial morphological analysis and quantifications (Fig. 3j-m) showed that both the intracellular mitochondrial and the total area were significantly decreased in the presence of L166P and M26I (Fig. 3k). Quantitative morphological analysis (Fig. 3l) demonstrated that the number of mitochondrial branches, total branch length, and branch junctions were significantly reduced in the L166P and M26I groups compared to the WT group. Furthermore, the mean branch length and the number of branch junctions per mitochondrion were significantly lower in the L166P group than in both the WT and M26I groups (Fig. 3m).

We also assessed the expression levels and stability of these related proteins (Fig. 3n-r). While endogenous DJ-1 expression remained unaltered (Fig. 3o), the expression of the overexpressed L166P mutant was significantly lower than that of WT and M26I (Fig. 3p-r). Consistent with previous results, the His-Myc tagged L166P exhibited obvious degradation bands (Fig. 3n). Simultaneously, we examined BNIP3 and BNIP3L expression (Fig. 3s). Further analysis in SH-SY5Y cells revealed that in the presence of L166P, the levels of monomeric BNIP3 (25–34 kDa, blue bars) were significantly higher compared to the WT and M26I groups (Fig. 3t). Additionally, its oligomers (~ 72 kDa, green bars) were also significantly elevated compared to the WT and M26I groups. For BNIP3L, no monomer was detected in SH-SY5Y cells (Fig. 3u). Overexpression of WT DJ-1, as well as the L166P and M26I mutants, significantly decreased the levels of the lower panel BNIP3L oligomers (green bars). Conversely, the L166P and M26I mutants significantly increased the levels of the upper panel BNIP3L oligomers (blue bars). Total BNIP3L levels showed a significant decrease only in the WT group, with no significant alterations observed between the mutant groups (Fig. 3v).

### AlphaFold3 guided modeling identifies candidate interaction regions among DJ-1, BNIP3, and BNIP3L

**Fig. 4:**
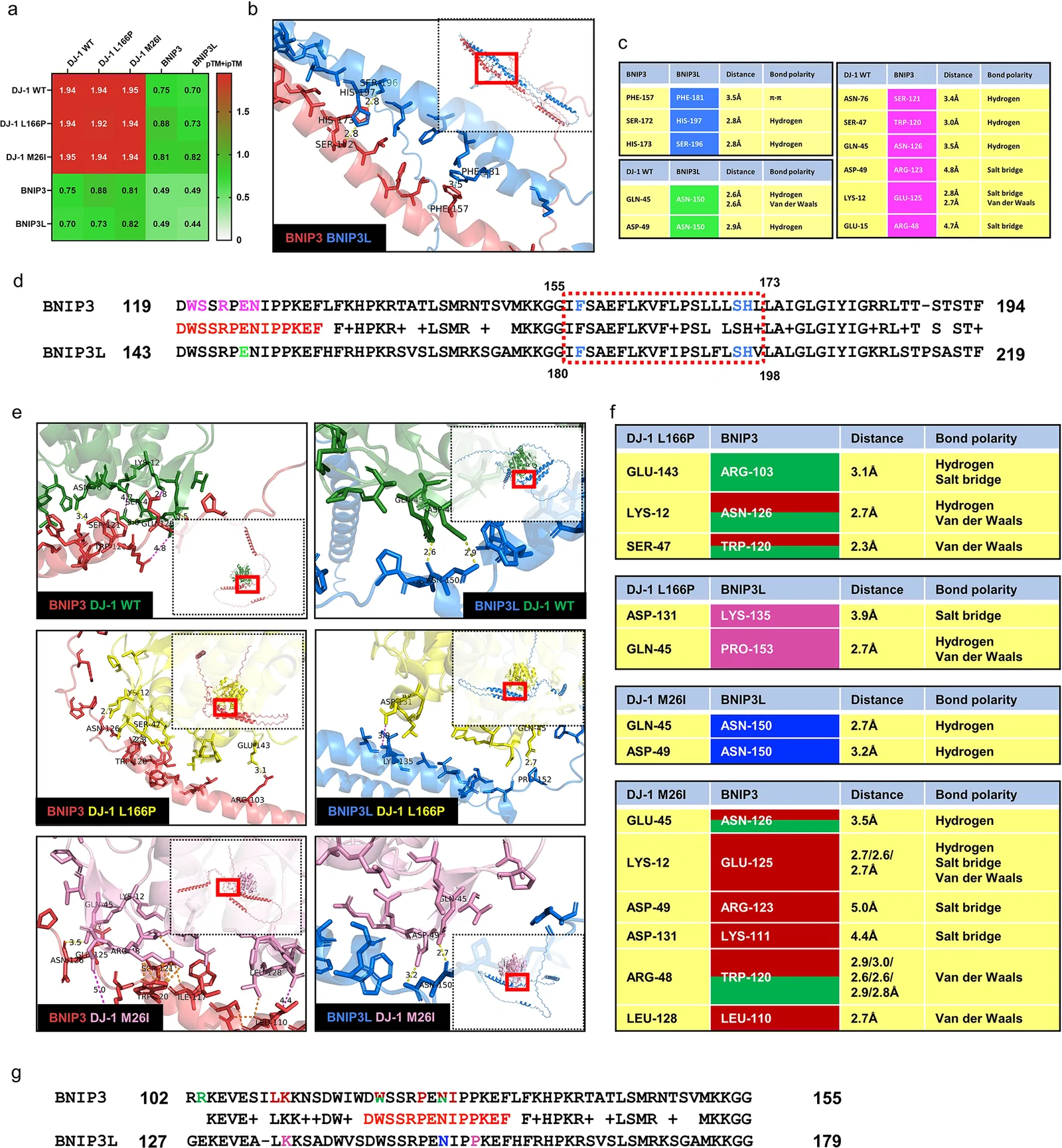
AlphaFold3 guided modeling of candidate interaction regions among DJ-1, BNIP3, and BNIP3L. **a** AlphaFold3 model confidence scores, expressed as the sum of pTM and ipTM, for the indicated DJ-1, BNIP3, and BNIP3L combinations. **b** AlphaFold3 generated structural model of the BNIP3-BNIP3L complex. **c** Detailed views of candidate interface residues and potential intermolecular contacts in the modeled BNIP3-BNIP3L, DJ-1 WT-BNIP3, and DJ-1 WT-BNIP3L complexes. **d** Primary sequence alignment of BNIP3 and BNIP3L showing candidate interface residues. Rose-red indicates candidate BNIP3 residues in the model with DJ-1 WT; green indicates candidate BNIP3L residues in the model with DJ-1 WT; sky-blue indicates candidate residues in the BNIP3–BNIP3L model; and the red box indicates the transmembrane region. **e** AlphaFold3 generated structural models of BNIP3 or BNIP3L with DJ-1 WT, L166P, or M26I. **f** Detailed views of candidate interface residues and potential intermolecular contacts in the modeled complexes containing DJ-1 L166P or M26I. **g** Primary sequence maps highlighting candidate interface residues. Green and red indicate candidate BNIP3 residues in models containing DJ-1 L166P and M26I, respectively; pink and blue indicate candidate BNIP3L residues in models containing DJ-1 L166P and M26I, respectively

To further elucidate the underlying mechanisms of BNIP3 alterations in SH-SY5Y cells, we performed protein complex prediction analysis for human-derived BNIP3, BNIP3L, and DJ-1 (WT, L166P, and M26I). We utilized predictive interface residues analysis by AlphaFold3 (AF3) to elucidate the physical interaction potential among human BNIP3, BNIP3L, and DJ-1 variants. Interaction probabilities were evaluated using the sum of pTM and ipTM scores, where > 0.5 indicates possible interaction and > 0.75 indicates high confidence [23]. DJ-1 is known to function as a dimer; as depicted in Fig. 4a, the sum of pTM and ipTM scores for DJ-1 WT, L166P, and M26I dimers were all > 1.9, indicating highly stable structures. Interestingly, while the predicted interaction score for the BNIP3-BNIP3L complex alone was less than 0.5, the interaction scores between DJ-1 (WT, L166P, or M26I) and either BNIP3 or BNIP3L were all above 0.7. Notably, the binding score between the DJ-1 L166P mutant and BNIP3 reached as high as 0.88.

Based on the analysis of contact interface residues within the predicted protein complexes, the interacting structural domains and specific binding sites between BNIP3 and BNIP3L are shown in Fig. 4b and c, respectively. These interaction domains map to residues 155–173 of BNIP3 and residues 180–198 of BNIP3L, which are highlighted in blue in the sequence alignment of BNIP3 and BNIP3L (Fig. 4d). Furthermore, the interaction domains of WT DJ-1 with BNIP3 and BNIP3L are presented in Fig. 4e, with the specific interacting sites and regions mapped onto the protein sequences of BNIP3L (green) and BNIP3 (pink) in Fig. 4c and d.

Similarly, the interacting domains of the DJ-1 L166P and M26I mutants with BNIP3 and BNIP3L are shown in Fig. 4e, with their corresponding binding amino acid sites and sequences detailed in Fig. 4f and g. Crucially, sequence analysis revealed that the key binding interfaces between BNIP3/BNIP3L and DJ-1 (WT, L166P, and M26I) primarily involve the specific amino acid sequence “DWSSRPENIPPKEF” (highlighted in red) and the C-terminal region of DJ-1. The C-terminal L166P mutation abrogates their interaction, while the N-terminal M26I mutation has no impact on binding.

### DJ-1 L166P shows reduced detectable binding to BNIP3

**Fig. 5:**
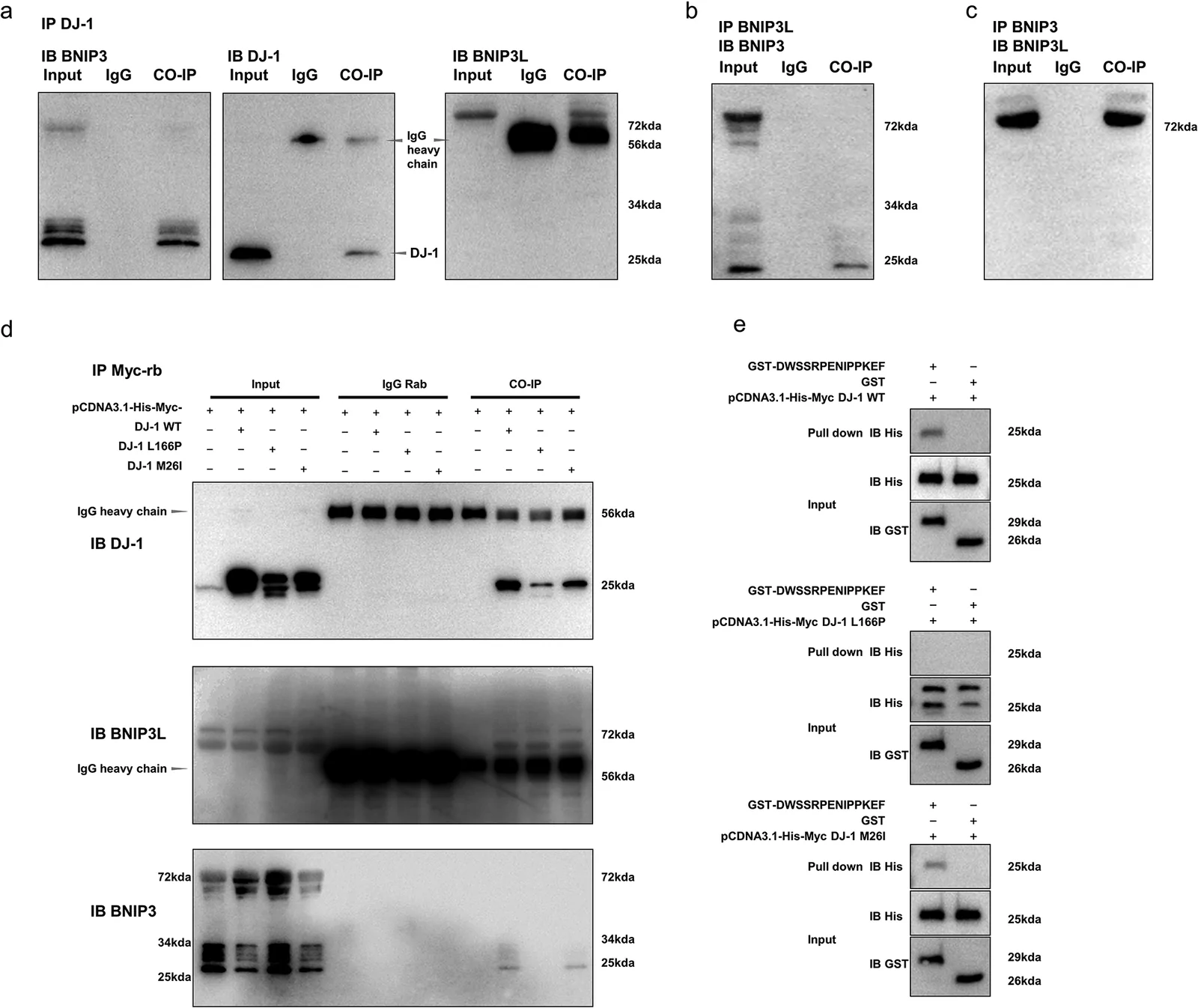
Co-immunoprecipitation and GST pull-down analyses of DJ-1, BNIP3, and BNIP3L associations in SH-SY5Y cells. **a-c** Endogenous co-immunoprecipitation assays in untreated SH-SY5Y cells. **a** Immunoprecipitation of DJ-1 followed by immunoblotting for BNIP3, DJ-1, and BNIP3L. **b** Immunoprecipitation of BNIP3L followed by immunoblotting for BNIP3. **c** Immunoprecipitation of BNIP3 followed by immunoblotting for BNIP3L. **d** Co-immunoprecipitation assays in SH-SY5Y cells stably expressing Myc-His tagged DJ-1 WT, L166P, or M26I. Lysates were immunoprecipitated using an anti-Myc antibody and analyzed by immunoblotting for DJ-1, BNIP3L, and BNIP3. **e** GST pull-down assay using bacterially expressed GST-tagged DWSSRPENIPPKEF peptide as bait and lysates from SH-SY5Y cells stably expressing His-tagged DJ-1 WT, L166P, or M26I as prey. Bound fractions were analyzed by immunoblotting for DJ-1

To explore the physiological interactions among DJ-1, BNIP3, and BNIP3L, we first performed Co-IP assays in untreated SH-SY5Y cells. As shown in Fig. 5a, endogenous DJ-1 was immunoprecipitated, followed by WB analysis for BNIP3, DJ-1, and BNIP3L. The results revealed positive interaction signals for both BNIP3 and BNIP3L with endogenous DJ-1. We further analyzed the interaction between BNIP3 and BNIP3L. Immunoprecipitation of BNIP3L followed by immunoblotting for BNIP3 detected a positive signal at 25 kDa (Fig. 5b). and reciprocal Co-IP of BNIP3 pulled down the BNIP3L (Fig. 5c), supporting a reciprocal interaction. We selected siDJ-1 #3 to suppress DJ-1 expression (Fig. S1a, b) and then performed reciprocal Co-IP for BNIP3 and BNIP3L. Under DJ-1 knockdown, the reciprocal BNIP3/BNIP3L interaction signal was detected (Fig. S1c). We also selected siBNIP3 #2 (Fig. S2a, b) and siBNIP3L #2 (Fig. S3a, b) to suppress BNIP3 and BNIP3L, respectively. As BNIP3 or BNIP3L protein levels decreased, the reciprocal BNIP3/BNIP3L interaction signal was lost or markedly reduced (Fig. S2c and Fig. S3c). These knockdown experiments support interaction and interdependence among DJ-1, BNIP3 and BNIP3L.

In cells overexpressing Myc tagged DJ-1 variants, BNIP3L co-precipitated with DJ-1 WT, L166P, and M26I (Fig. 5d, upper and middle panels). However, while BNIP3 robustly co-precipitated with DJ-1 WT and weakly with M26I, it failed to interact with the L166P mutant (Fig. 5d, lower panel). To examine whether the reduced binding signal was related to L166P instability, we used the proteasome inhibitor MG132 as a condition that partially limits L166P degradation. MG132 partially restored L166P protein levels (Fig. S4a, b), but it did not restore detectable BNIP3 binding in the Co-IP assay (Fig. S4c). These findings suggest that reduced BNIP3 binding is not solely explained by low L166P abundance.

Given that BNIP3 failed to co-immunoprecipitate with DJ-1 L166P in cells, we conducted an in vitro GST pull-down assay using the conserved peptide “DWSSRPENIPPKEF” which analyzed in last result. Consistent with the pull-down results (Fig. 5e), the GST tagged peptide successfully pulled down His-tagged DJ-1 WT and M26I, but failed to pull down the L166P mutant. To test whether the functional site of W2 and N8 contribute to binding, a mutant peptide was replaced with alanine to reduce side chain hydrogen bond and hydrophobic contributions while minimizing disruption of the peptide backbone. The mutant peptide DASSRPEAIPPKEF did not efficiently pull down His-tagged DJ-1 WT, L166P, or M26I under the tested conditions (Fig. S5). These GST pull-down data support DWSSRPENIPPKEF peptide interface region to DJ-1 associated binding.

### GRP75 and BNIP3 depletion alter TEM based ER-mitochondria proximity measurements in DJ-1 variant expressing cells

**Fig. 6:**
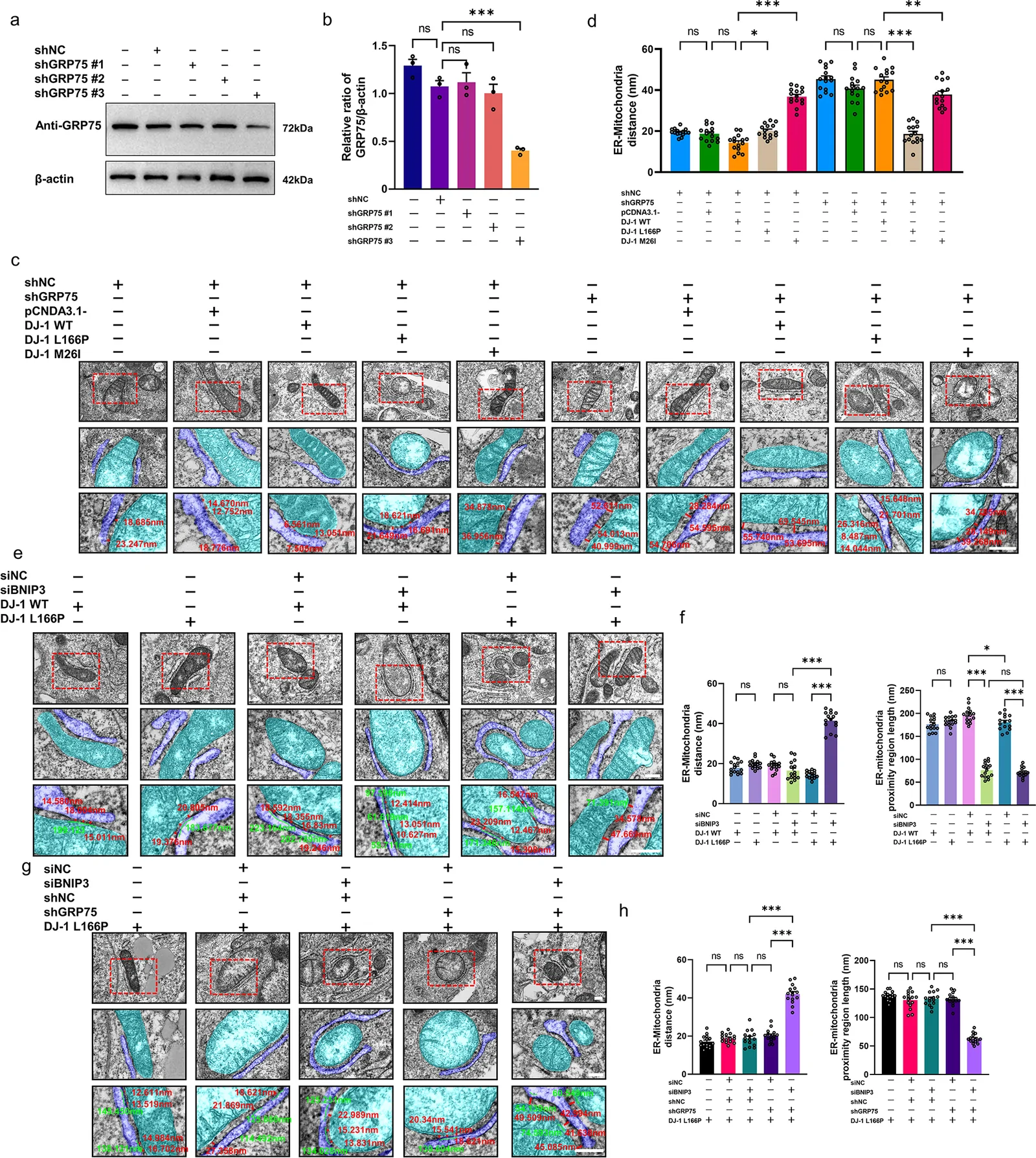
TEM based ER-mitochondria proximity measurements following GRP75 and BNIP3 depletion in SH-SY5Y cells. **a**,** b** Western blotting and densitometric analysis confirming shRNA-mediated GRP75 knockdown. **c** Representative TEM images of ER-mitochondria proximity regions in SH-SY5Y cells stably expressing shNC or shGRP75 and transiently expressing DJ-1 WT, L166P, or M26I. **d** Quantification of the perpendicular distance between the ER membrane and the outer mitochondrial membrane under the conditions shown in (**c**). **e** Representative TEM images of ER-mitochondria proximity regions in cells stably expressing DJ-1 WT or L166P and transiently transfected with siNC or siBNIP3. **f** Quantification of ER-mitochondria distance and proximity region length under the conditions shown in (**e**). **g** Representative TEM images of ER-mitochondria proximity regions in cells stably expressing DJ-1 L166P and transfected with shGRP75 together with either siNC or siBNIP3. **h** Quantification of ER-mitochondria distance and proximity region length under the conditions shown in g. Mitochondria and the ER were pseudocolored teal and lavender, respectively. Red lines indicate the perpendicular distance between the ER membrane and the outer mitochondrial membrane, and green lines indicate the continuous segments used to measure proximity-region length. Scale bar = 200 nm. Data are presented as mean +/- SEM, *N* = 15. *P < 0.05, **P < 0.01, ***P < 0.001; ns,* not significant*

Previous studies have implicated DJ-1 in the regulation of the canonical IP3R3-GRP75-VDAC1 complex and ER-mitochondria communication. To examine local ER-mitochondria ultrastructural proximity under reduced GRP75 expression, we used shRNA to knock down GRP75. Western blotting confirmed that the shGRP75 #3 construct markedly reduced GRP75 protein levels and was therefore selected for subsequent experiments (Fig. 6a, b).

We next used TEM to assess ER-mitochondria proximity in cells expressing DJ-1 WT, L166P, or M26I under sh-NC or shGRP75 conditions. Representative images are shown in Fig. 6c. Mitochondria and the ER were pseudocolored teal and lavender, respectively, and red lines indicate the measured perpendicular distance between the ER membrane and the outer mitochondrial membrane. Under sh-NC conditions, the mean ER-mitochondria distance was 14.25 ± 1.04 nm in the DJ-1 WT group, 20.16 ± 0.81 nm in the L166P group, and 36.81 ± 1.09 nm in the M26I group (Fig. 6c, d). Following GRP75 knockdown, the mean ER-mitochondria distance increased significantly to 45.04 ± 1.42 nm in the DJ-1 WT group. The corresponding mean distances were 18.66 ± 1.26 nm in the L166P group and 37.85 ± 1.60 nm in the M26I group. Thus, GRP75 knockdown was associated with distinct DJ-1 variant dependent patterns of ER-mitochondria distance.

Because our preceding experiments showed increased BNIP3 monomer abundance in L166P expressing cells, we next examined whether BNIP3 depletion affected TEM based ER-mitochondria proximity measurements. Cells stably expressing DJ-1 WT or L166P were treated with siBNIP3 and analyzed by TEM (Fig. 6e). BNIP3 depletion increased the ER-mitochondria distance from 14.39 ± 0.46 nm to 41.46 ± 1.21 nm in L166P groups. In addition, siBNIP3 significantly reduced the length of the ER-mitochondria proximity region in both the DJ-1 WT from 196.897 ± 4.267 nm to 76.627 ± 4.185 nm and L166P groups from 180.61 ± 4.21 nm to 71.67 ± 2.60 nm, as quantified in Fig. 6f. Green lines in the representative images indicate the segments used to measure the length of the proximity regions.

We next examined the effect of additional BNIP3 depletion under GRP75 knockdown conditions in cells stably expressing DJ-1 L166P. Compared with the shGRP75 plus siNC condition, the shGRP75 plus siBNIP3 condition showed an increase in the mean ER-mitochondria distance from 20.23 ± 0.85 nm to 42.05 ± 1.27 nm and a reduction in the mean proximity region length from 132.39 ± 2.80 nm to 62.76 ± 2.22 nm (Fig. 6g, h). Collectively, these TEM based analyses identified changes in ER-mitochondria distance and proximity region length within the analyzed TEM sections following GRP75 and/or BNIP3 depletion.

Collectively, these TEM based analyses identified changes in ER-mitochondria distance and proximity region length within the analyzed TEM sections following GRP75 and/or BNIP3 depletion.

### Proximity associated imaging readouts and Fluo-4 responses in DJ-1 variant expressing cells under BNIP3 depleted conditions

Because BNIP3 depletion altered TEM based ER-mitochondria proximity measurements, we next examined proximity associated colocalization readouts, selected ER and mitochondrial protein levels, and global intracellular Fluo-4 Ca^2+^ responses in DJ-1 variant expressing cells under siBNIP3 conditions. COX4/PDI and ERp72/VDAC1 colocalization were quantified as ER-mitochondria proximity associated imaging readouts. Because the nanometer scale separation between ER and mitochondrial membranes is below the resolution limit of conventional fluorescence microscopy, these colocalization coefficients were interpreted as indicators of regional organelle proximity rather than direct measurements of membrane contacts.

**Fig. 7:**
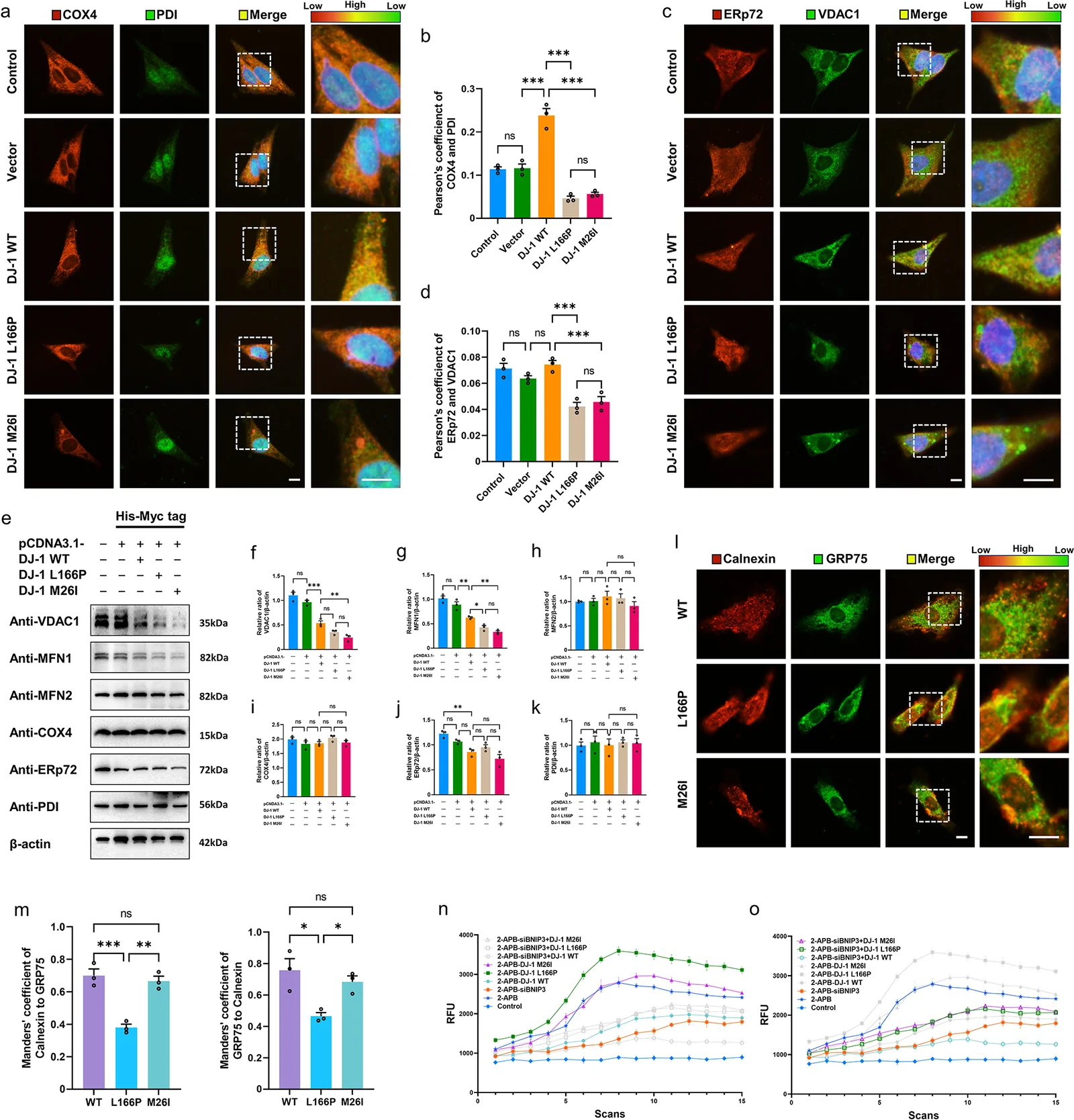
BNIP3 depletion alters ER-mitochondria proximity associated readouts and Fluo-4 responses in DJ-1 variant expressing cells. Assays were performed in SH-SY5Y cells stably expressing DJ-1 WT, L166P, or M26I and transiently transfected with siBNIP3. **a** Representative fluorescence images showing COX4 (red) and PDI (green). Yellow regions in the merged images indicate overlapping red and green fluorescence signals. **b** PCC analysis for COX4 and PDI colocalization. **c** Representative fluorescence images showing ERp72 (red) and VDAC1 (green). **d** PCC analysis for ERp72 and VDAC1 colocalization. **e** Western blot analysis of VDAC1, MFN1, MFN2, COX4, ERp72, and PDI. **f-k** Quantitative densitometric analysis of the proteins in (**e**). **l** Representative fluorescence images showing Calnexin (red) and GRP75 (green).**m** Manders’ colocalization coefficients. M1 represents the fraction of the Calnexin signal overlapping with GRP75, and M2 represents the fraction of the GRP75 signal overlapping with Calnexin. **n**,** o** Fluo-4 RFU time course responses following 2-APB stimulation. Panels n and o present the same dataset with different visual emphasis to facilitate comparison among the indicated groups. Data are presented as mean +/- SEM, *N* = 3. *P < 0.05, **P < 0.01, ***P < 0.001; *ns*,* not significant*

We first evaluated the colocalization of the inner mitochondrial membrane marker COX4 and the ER marker PDI under siBNIP3 conditions (Fig. 7a). Under siBNIP3 conditions, the COX4/PDI PCC was significantly higher in the DJ-1 WT group than in the L166P and M26I groups. (Fig. 7b). We next examined colocalization between the ER marker ERp72 and the outer mitochondrial membrane marker VDAC1 (Fig. 7c). Although the overall PCC values were below 0.1, under siBNIP3 conditions, the ERp72/VDAC1 PCC did not differ significantly between the control and DJ-1 WT groups but was significantly lower in the L166P and M26I groups than in the DJ-1 WT group. (Fig. 7d).

We then examined the protein levels of these and other ER and mitochondria associated markers (Fig. 7e). Under siBNIP3 conditions, VDAC1 and MFN1 levels were significantly lower in L166P- and M26I-expressing cells than in DJ-1 WT-expressing cells (Fig. 7f, g). In contrast, COX4, MFN2, ERp72, and PDI levels did not show significant changes under the corresponding conditions (Fig. 7h-k).

Because VDAC1 and MFN have been linked to cellular Ca^2+^ regulation [24, 25], we also examined the colocalization of GRP75, also known as mtHSP70, with the ER marker Calnexin. The spatial relationship between these proteins has been used as an imaging readout associated with ER-mitochondria Ca^2+^ signaling [26]. As shown in Fig. 7l and m, under siBNIP3 conditions, both M1, representing the fraction of Calnexin signal overlapping with GRP75, and M2, representing the fraction of GRP75 signal overlapping with Calnexin, were significantly lower in the L166P group than in the DJ-1 WT and M26I groups.

We next used Fluo-4 fluorescence measured with a microplate reader as an indirect readout of global intracellular Ca^2+^ responses [27]. Cells were stimulated with 10 µM 2-APB, and the resulting fluorescence changes were recorded over time [28, 29]. Figure 7n and o present the same RFU time course dataset, with different groups emphasized to facilitate comparison. Without BNIP3 knockdown, L166P expressing cells showed a significantly greater 2-APB evoked Fluo-4 response than DJ-1 WT expressing cells, whereas the M26I group did not differ significantly from the WT group (Fig. 7n), indicating different response patterns for the two variants under this condition. Following BNIP3 depletion, neither the L166P nor the M26I group differed significantly from the WT group in the Fluo-4 time course response (Fig. 7o). The response amplitude, expressed as ΔFmax, and the integrated response, expressed as ΔAUC, are shown in Supplementary Fig. S6a and b, respectively. The time required to reach the peak response was comparable among the groups (Supplementary Fig. S6c), and the relative peak response normalized to the fully negative control is shown in Supplementary Fig. S6d. Based on this analysis, siBNIP3 reduced the relative response in the WT, L166P, and M26I groups by 40.7%, 76.7%, and 42.5%, respectively.

Collectively, BNIP3 depletion was accompanied by distinct, DJ-1 variant dependent changes in ER-mitochondria proximity associated imaging readouts, selected mitochondrial protein levels, and global intracellular Fluo-4 Ca^2+^ responses.

## Discussion

Hereditary early-onset Parkinson’s disease caused by *PARK7* mutations is an autosomal recessive disorder. Among the known homozygous mutations, L166P, M26I, and E163K confer a higher pathogenic risk and result in more severe clinical symptoms compared to heterozygous mutations (such as D149A, E64D, and A39S). The E163K mutation reported in OMIM is accompanied by an 18-base pair duplication (168-185dup) in the DJ-1 promoter region, presenting with complex phenotypes including ALS and cognitive impairment alongside PD symptoms. To strictly isolate the effects of canonical missense mutations on protein structure and function, while avoiding the confounding influences of promoter variations and complex atypical phenotypes, our study exclusively focused on the L166P (C-terminal) and M26I (N-terminal) mutations. The instability of the DJ-1 L166P mutant is well-documented, largely attributed to the disruption of its C-terminal α7 helix, loss of dimerization capacity, and accelerated proteasome mediated degradation [30]. The L166P variant is known to destabilize DJ-1 protein structure, and our data are consistent with reduced L166P abundance in both PC-12 and SH-SY5Y cell models. The MG132 experiment further indicates that partial restoration of L166P protein abundance does not restore detectable BNIP3 binding under the tested Co-IP conditions. These findings support the interpretation that the L166P phenotype reflects both protein instability and altered interaction behavior. L166P is located in the C-terminal region of DJ-1 and is associated with reduced detectable BNIP3 binding, whereas M26I is located in the N-terminal region and does not show the same loss of BNIP3 binding. Because L166P also affects DJ-1 stability and conformation, C-terminal L166P containing region is important for DJ-1 structural stability and BNIP3 binding competence. M26I appeared more stable than L166P in the expression assays but was not functionally identical to stable DJ-1 WT in the colocalization and proximity associated analyses. DJ-1 variants can differ in both protein stability and assay specific interaction or proximity phenotypes.

Rat Bnip3l and human BNIP3L differ at only six amino acid positions, and the mid-to-posterior segments of human BNIP3 and rat Bnip3l are highly conserved. Consistent with our structural predictions (pTM + ipTM scores) and previous reports that BNIP3 and BNIP3L can form homo- or hetero-oligomers [14, 16, 31]. Together, the AF3 predictions, Co-IP assays, GST pull-down assays, MG132 experiment, and mutant peptide pull-down support a model in which DJ-1 variants differentially affect BNIP3/BNIP3L associated interaction networks. The reciprocal knockdown experiments further support interdependence among DJ-1, BNIP3, and BNIP3L in this experimental system. These data nominate BNIP3/BNIP3L associated complexes as candidate contributors to ER-mitochondria proximity phenotypes.

The predicted interaction interfaces encompass the BRCT and MAPK domains of BNIP3 (residues 155–173) and the MAPK domain of BNIP3L (residues 180–198), both of which overlap significantly with their transmembrane (TM) domains [32]. Although the N-terminus of rat Bnip3 differs considerably from its human counterpart, our Co-IP and co-localization data confirmed robust interactions between both human BNIP3-BNIP3L and rat Bnip3-Bnip3l, suggesting that these N-terminal variations do not dictate their binding capacity. Interestingly, while the M26I mutation did not significantly reduce BNIP3-BNIP3L co-localization, L166P drastically diminished it. AlphaFold3 interface analysis (Fig. 4e–g) identified the proline-rich sequence DWSSRPENIPPKEF and its adjacent region as a candidate interaction region for DJ-1 (WT, L166P, M26I) with BNIP3/BNIP3L. But there is no evidence of the formation of a triple protein complex as shown in Fig. S7. The M26I mutant exhibits the highest number of predicted binding sites with BNIP3, which may explain its higher Pearson correlation coefficient with BNIP3 in SH-SY5Y cells compared to L166P (Fig. 3f, g).

Previous structural studies have established that the TM domains of MFN1/2 are essential for their homo/hetero-dimerization [33], our results indicate that in the L166P case, the decrease of MFN1 may due to the fact that L166P will aggregate and damage the mitochondria [34], thereby mediating the entry of mitochondria into the autophagy pathway mediated by PINK1/Parkin [35], and subsequently leading to the reduction of outer membrane protein MFN1 [36]. In summary, the stability of the TM domain in the TOM complex maintains the structural stability of MFN [37–39]. It is highly probable that the BNIP3-BNIP3L oligomerization interface relies on these structurally predicted TM-encompassing domains (Fig. 4d, red frame). The TM domains of BNIP3 and BNIP3L oligomerization likely establishes a structural scaffold that bridges opposing mitochondrial outer membranes during mitophagy, analogous to the TM-dependent dimerization of MFN1/2 that physically tethers mitochondria during fusion. However, whether this TM-centric interaction directly recruits ER-resident proteins or merely provides passive membrane anchoring remains to be determined. Due to the high homology of these 18 amino acids (containing only two synonymous and one missense mutation) and their indispensable role in outer mitochondrial membrane targeting, selectively mutating these regions to test BNIP3-BNIP3L stability without disrupting their basal subcellular localization remains technically challenging. Nevertheless, the AF3 derived predictions and Co-IP results nominate the TM region and its immediate flanking residues as candidate interaction regions for further investigation.

DJ-1 is known to interact with and stabilize the canonical IP3R3-Grp75-VDAC1 MAM complex. In this study, we identified DJ-1 associations with BNIP3 and BNIP3L. Endogenous BNIP3 is inserted into both the outer mitochondrial membrane (OMM) and the ER; however, its rapid clearance via the ER to lysosome pathway results in a predominantly mitochondrial steady state localization. Blocking its ER insertion increases BNIP3 dimerization at the OMM and subsequently triggers mitophagy [40]. Similarly, BNIP3L is a “BH3 only like” protein localized to both the OMM and ER, implicated in apoptosis and necroptosis [41]. Given their dual OMM/ER localization, we explored whether the BNIP3-BNIP3L heterodimer might contribute to ER-mitochondria membrane organization.

To assess ER-mitochondria ultrastructural proximity under reduced GRP75 expression, we depleted GRP75 and examined the resulting TEM phenotypes in DJ-1 WT, L166P, and M26I expressing cells. Under these conditions, the L166P group showed shorter ER-mitochondria distances than the WT and M26I groups within the analyzed TEM sections. Because TEM provides static, two-dimensional measurements, these observations should be interpreted as local ultrastructural proximity differences rather than evidence of whole cell or dynamic reorganization.

In light of the reduced DJ-1 BNIP3 interaction observed for L166P and the altered BNIP3 related phenotypes in this study, it is possible that changes in the DJ-1/BNIP3/BNIP3L interaction network may be associated with the proximity related measurements observed after GRP75 perturbation. However, the present data do not establish whether this reflects a compensatory mechanism, altered membrane organization, or another indirect consequence of the perturbation.

The different TEM phenotypes observed under transient and stable DJ-1 expression should also be interpreted in the context of the distinct experimental paradigms. The more pronounced distance phenotype observed under transient L166P expression may reflect acute proteotoxic or mitochondrial stress, whereas stable selection and prolonged culture may permit adaptive or compensatory changes that attenuate the corresponding phenotype. This post hoc interpretation was not directly tested, and differences in expression level, perturbation sequence, or cellular selection may also contribute.

Prior studies have reported that GRP75 knockdown attenuates DJ-1 overexpression induced mitochondrial translocation during hypoxic preconditioning [42], and that DJ-1 overexpression rescues p53 induced reduction in ER-mitochondria contact sites [6], suggesting complex regulatory interplay between these proteins in modulating ER-mitochondria interactions. Regarding the additional ER and mitochondrial marker analyses, under siBNIP3 conditions, PDI/COX4 colocalization was higher in DJ-1 WT expressing cells than in L166P and M26I expressing cells, whereas ERp72/VDAC1 colocalization was lower in the L166P and M26I groups than in the WT group. These findings indicate DJ-1 variant dependent differences in the regional spatial overlap of these marker pairs within the resolution limits of conventional fluorescence microscopy. Although the direction of these changes resembles proximity associated phenotypes previously reported after perturbation of the canonical IP3R3-GRP75-VDAC1 axis, the present colocalization data do not establish structural disruption or demonstrate that the same underlying mechanism is involved.

Our GST pull-down assays using the DWSSRPENIPPKEF peptide provided complementary evidence of DJ-1 variant dependent peptide associated binding under the tested conditions. His tagged DJ-1 WT and M26I were detected in the peptide pull-down fractions, whereas no corresponding L166P signal was detected under the tested conditions, consistent with the reduced BNIP3 association observed by Co-IP. This indicates that WT DJ-1 normally binds the C-terminus of this peptide, potentially restricting or regulating BNIP3/BNIP3L oligomerization. The L166P mutation abolishes this regulatory binding, allowing unregulated BNIP3-BNIP3L dimerization. Furthermore, this interaction interface overlaps with the binding domain for TRAF2/6 [32]. Evidence shows that L166P can bind TRAF6 accumulate into insoluble cytoplasmic aggregates [43]. The inability of L166P to bind BNIP3 might leave BNIP3 free to interact aberrantly with TRAF, thereby modulating TNF signaling. Since TRAF2 depletion inhibits TNF-α induced cell death but exacerbates oxidative stress induced apoptosis [44], this crosstalk elegantly explains why L166P induced cytotoxicity is predominantly linked to oxidative stress [45] and mitochondrial damage as observed in this study.

Previous studies have combined Calnexin/GRP75 colocalization measurements with mitochondrial Ca^2+^ indicators to investigate ER-mitochondria-associated Ca^2+^ signaling [26]. In the present study, Manders’ coefficients were used to quantify the directional fractional overlap of the Calnexin and GRP75 fluorescence signals. Under siBNIP3 conditions, both M1 and M2 were lower in L166P expressing cells than in DJ-1 WT and M26I expressing cells, indicating reduced directional fractional overlap of the Calnexin and GRP75 signals. Previous studies have shown that knocking down BNIP3 blocks full length BNIP3 mediated ER to mitochondria calcium transfer, restoring calcium homeostasis [46]. We additionally measured the 2-APB-evoked Fluo-4 RFU response as a global intracellular Ca²⁺-related fluorescence readout. BNIP3 depletion reduced the relative Fluo-4 response including RFU time course, ΔFmax, and ΔAUC in the DJ-1 WT, L166P, and M26I expressing groups. siBNIP3 reduced the 2-APB evoked Fluo-4 response. The largest proportional reduction was observed in the L166P expressing group. These findings show that the global intracellular Fluo-4 response was sensitive to BNIP3 depletion in all three DJ-1 expressing groups, with the greatest proportional change observed in L166P expressing cells.

Taken together, the morphological analyses, proximity associated imaging measurements, and global intracellular Ca^2+^ responses provide complementary evidence that DJ-1 variants are associated with BNIP3/BNIP3L related ER-mitochondria phenotypes. Nevertheless, these findings should be interpreted in light of the methodological limitations of the approaches used. Conventional diffraction limited fluorescence microscopy cannot resolve the nanometer scale separation between ER and mitochondrial membranes. Accordingly, the colocalization coefficients reported here represent regional spatial overlap rather than direct measurements of membrane proximity or dynamic ER-mitochondria organization. TEM provides nanometer scale information on ER-mitochondria distance and proximity region length, but it is restricted to static, two-dimensional observations of selected sections and cannot capture the temporal behavior or whole cell three-dimensional distribution of these structures. Similarly, Fluo-4 measurements reflect global intracellular Ca^2+^ responses and do not directly resolve directional Ca^2+^ transfer between the ER and mitochondria. Therefore, the proposed DJ-1/BNIP3/BNIP3L interaction network should be regarded as a candidate model associated with the observed phenotypes rather than a demonstrated autonomous tethering or functional coupling mechanism.

Genetically encoded proximity reporters would provide an important next step for evaluating this model. Split-GFP based contact site sensors (SPLICS) reconstitute fluorescence when reporter fragments targeted to opposing organelle membranes are brought within defined proximity ranges [47]. Short and long range ER-mitochondria SPLICS variants detect membrane juxtapositions over approximately 8–10 nm and 40–50 nm, respectively, whereas P2A based constructs permit balanced expression of the two reporter components and puncta based analysis in fixed or living cells. However, SPLICS remains a proximity based readout rather than direct evidence of molecular tethering or functional coupling, and the potentially limited reversibility of split-GFP complementation should be considered when interpreting real-time contact formation and dissociation [48, 49]. Applying these reporters to cells expressing DJ-1 WT, L166P, or M26I under GRP75 and BNIP3 perturbation could help determine whether the observed phenotypes are accompanied by changes in the abundance, spatial distribution, or distance range of ER-mitochondria proximity regions across the cellular volume. Combining such reporters with organelle targeted Ca^2+^ sensors, three-dimensional live cell imaging, loss of function and rescue experiments, and biochemical analysis of MAM enriched fractions would provide a more rigorous assessment of the spatial and functional relevance of the DJ-1/BNIP3/BNIP3L associated interaction network.

Several aspects of the present study warrant further investigation. Quantitative ER morphology was not assessed in the present study, leaving unresolved whether ER structural remodeling accompanies the observed mitochondrial and proximity associated phenotypes. Some experiments relied on transient or stable overexpression, peptide binding assays, or sequential knockdown treatments. In particular, the different TEM phenotypes observed under transient and stable DJ-1 expression may reflect acute proteotoxic or mitochondrial stress during transient L166P expression and adaptive or compensatory responses during stable selection and prolonged culture. This interpretation is post hoc and was not directly tested, and differences in expression level, perturbation sequence, or cellular selection may also contribute. Future studies under endogenous expression conditions will therefore be important for defining the physiological stoichiometry, subcellular localization, and functional relevance of the identified interactions.

In conclusion, cross species sequence comparison and AlphaFold3 guided modeling identified candidate BNIP3 and BNIP3L associated interaction regions, with complementary protein and peptide level evidence provided by co-immunoprecipitation and GST pull-down assays. The PARK7 L166P and M26I variants were associated with distinct DJ-1/BNIP3/BNIP3L interaction profiles, mitochondrial morphology phenotypes, ER-mitochondria proximity associated readouts, and global intracellular Fluo-4 Ca^2+^ responses. L166P showed marked instability and reduced BNIP3 binding, whereas M26I was comparatively stable but differed from DJ-1 WT in selected assays. Together, these findings support a candidate DJ-1/BNIP3/BNIP3L interaction network associated with ER-mitochondria related phenotypes under GRP75 or BNIP3 perturbation. More direct proximity based and organelle specific approaches will be required to establish the spatial and functional significance of these associations and to determine how they participate in PD pathogenesis.

## Supplementary Information

Below is the link to the electronic supplementary material.

Supplementary Material 1

Supplementary Material 2

## Data Availability

Data can be made available from the corresponding author upon reasonable request.
