# Supplementary material for: Effects of homozygous PARK7 gene mutations L166P and M26I on BNIP3/BNIP3L interactions and ER-mitochondria proximity
Source: Biol Direct. 2026 Aug 5;21:175. doi: 10.1186/s13062-026-00928-8 (PMC13563861; doi:10.1186/s13062-026-00928-8)

Effects of homozygous PARK7 gene mutations L166P and M26I on BNIP3/BNIP3L interactions and ER-mitochondria proximity

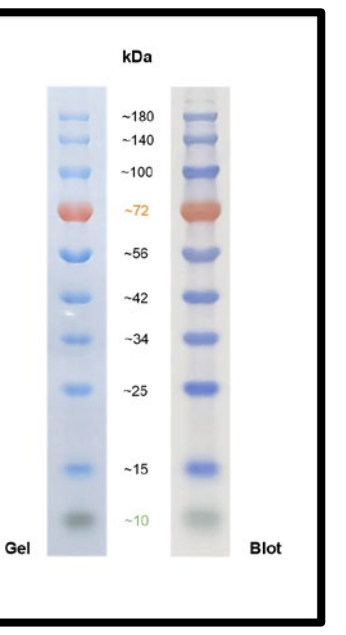

Figure 1e

Short Time

Long Time

DJ-1

GAPDH

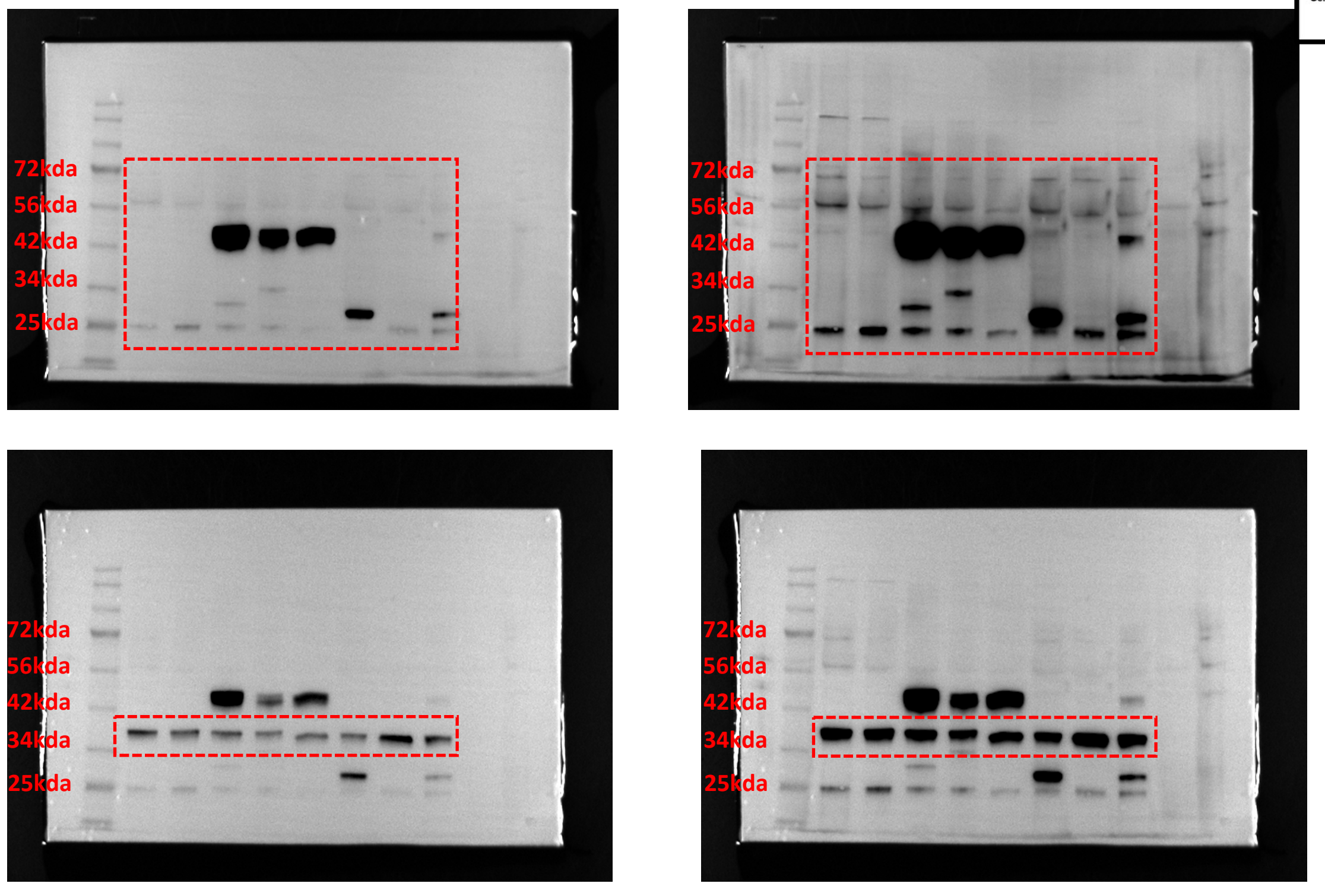

Figure 1 f g

Short Time

Long Time

DJ-1

β-actin

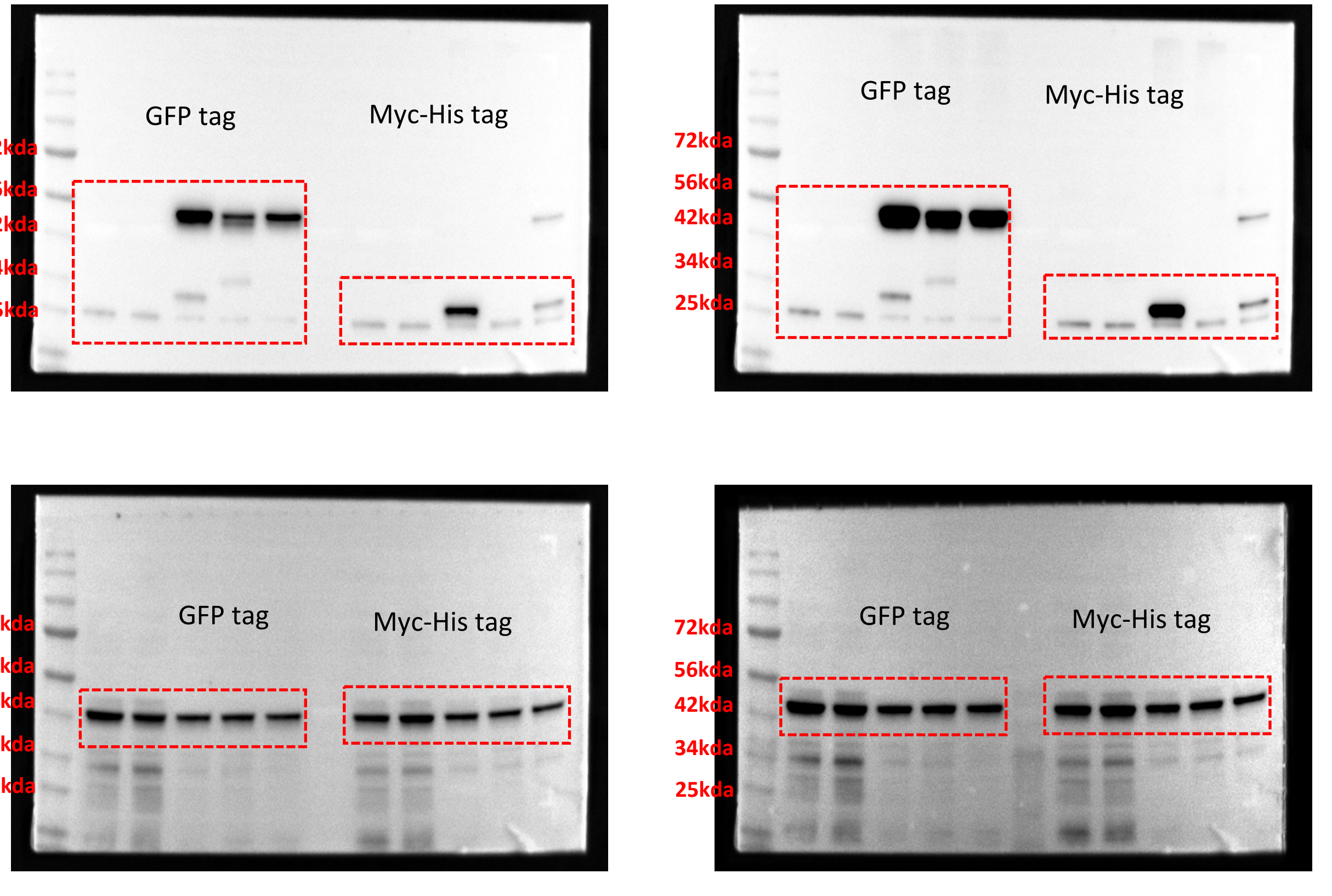

Figure 1j

His

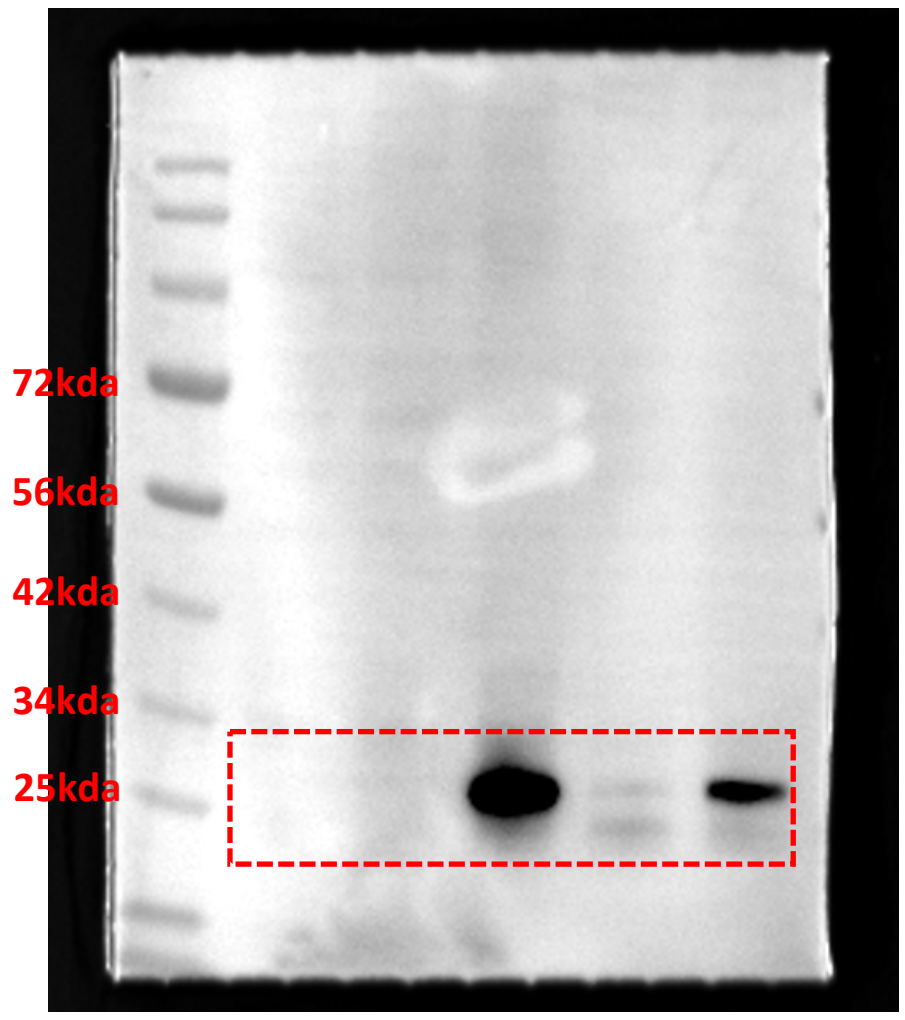

GFP

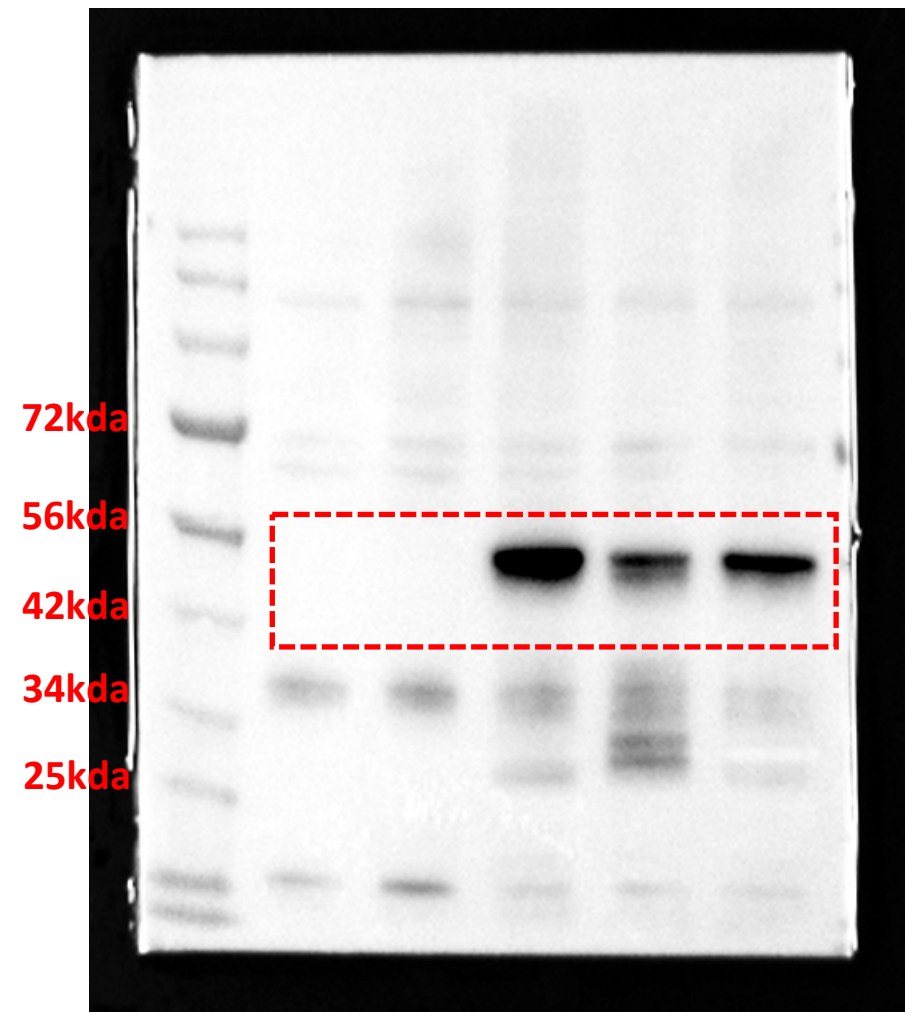

Myc

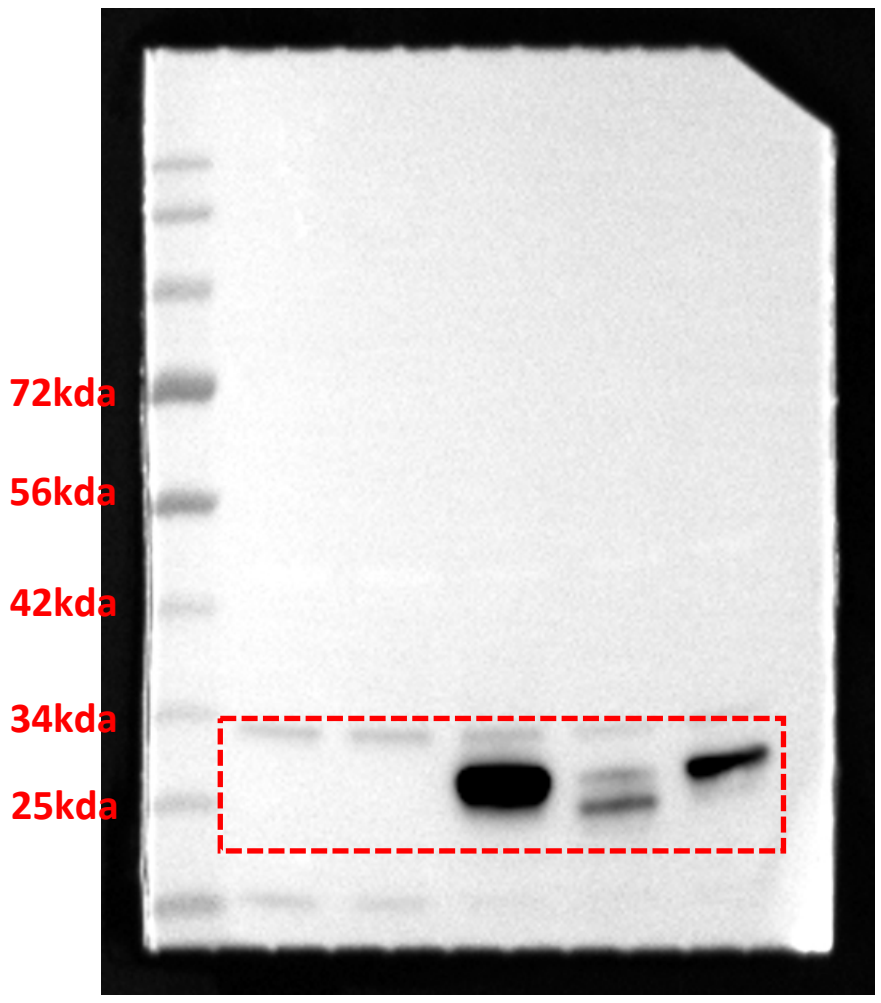

β-actin

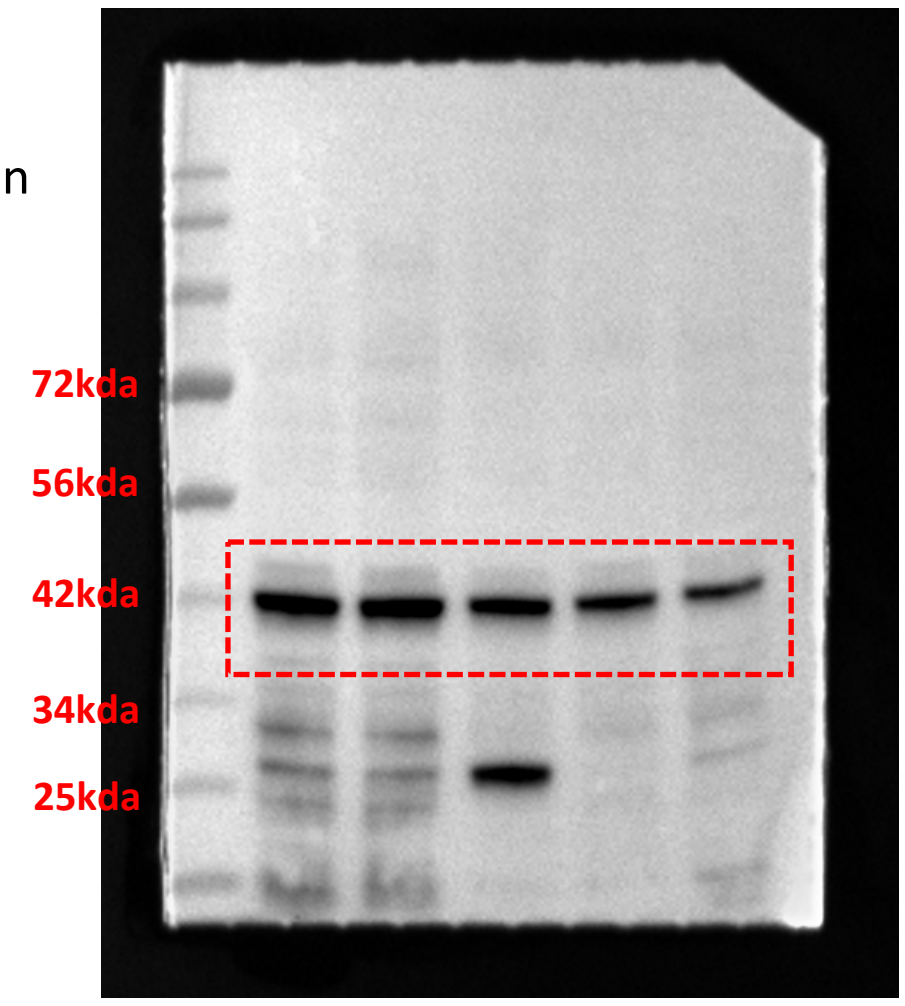

Figure 1n

Bnip3

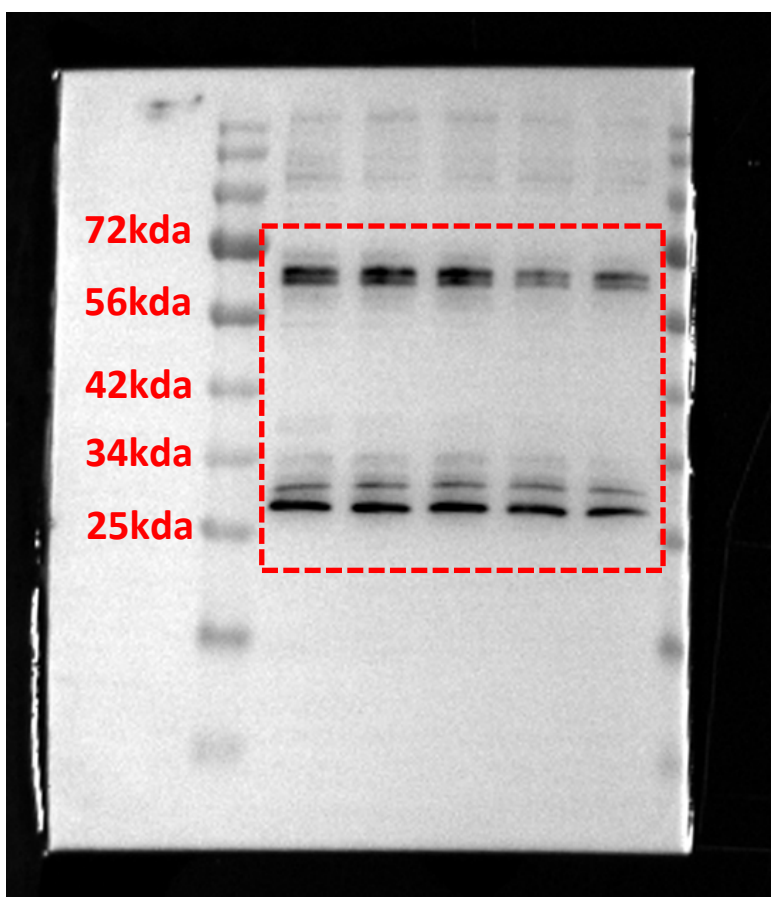

Bnip3l

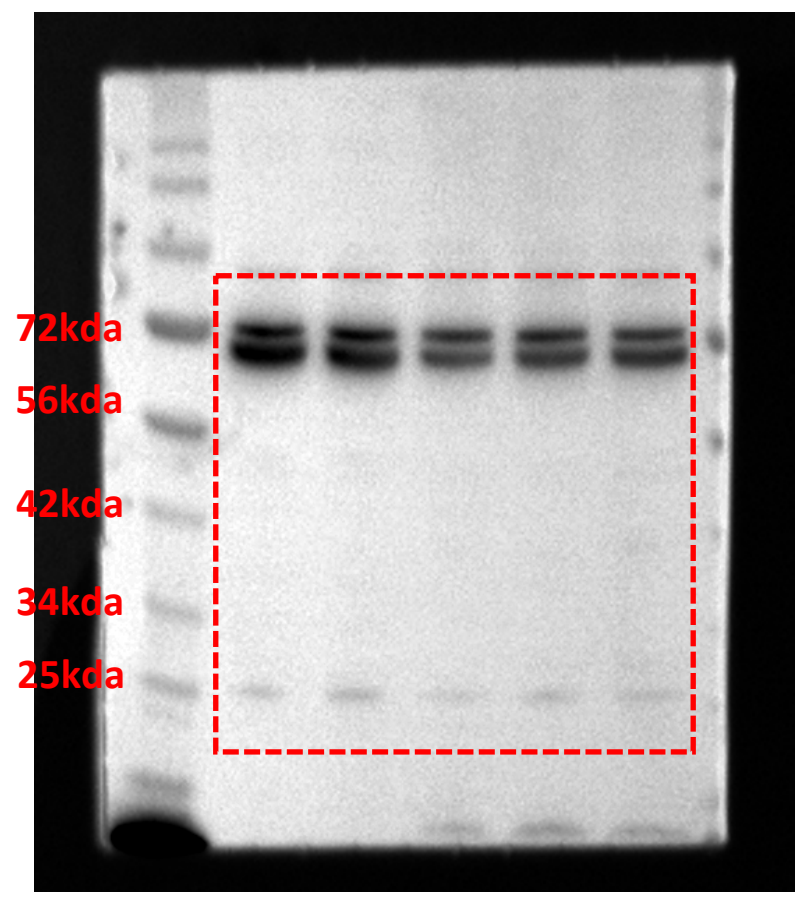

Mfn2

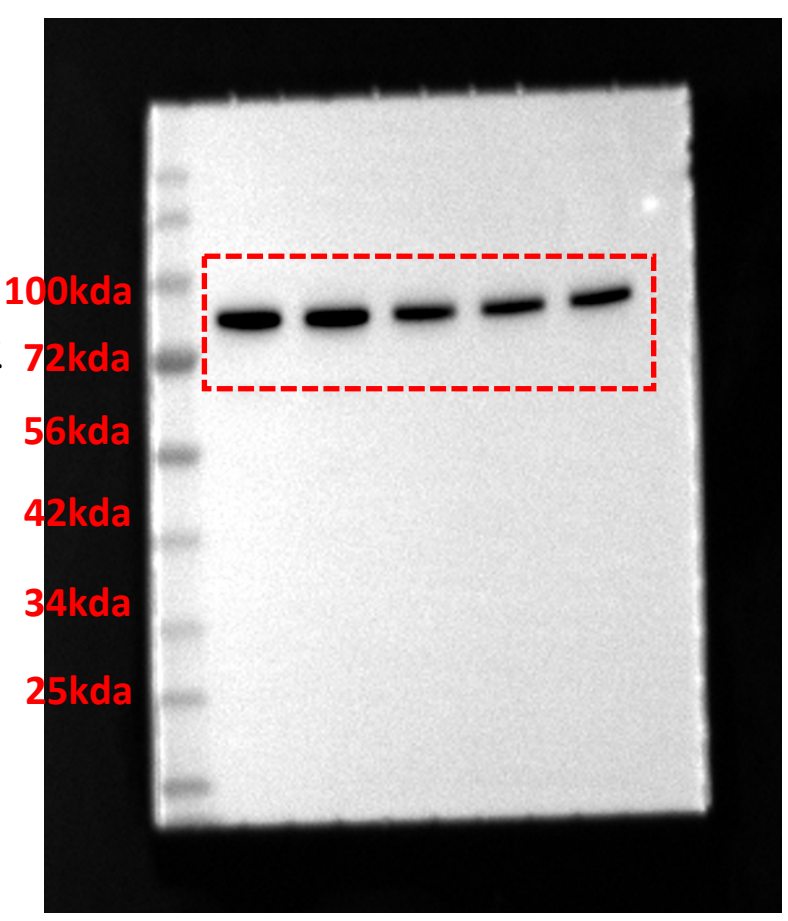

Mfn1

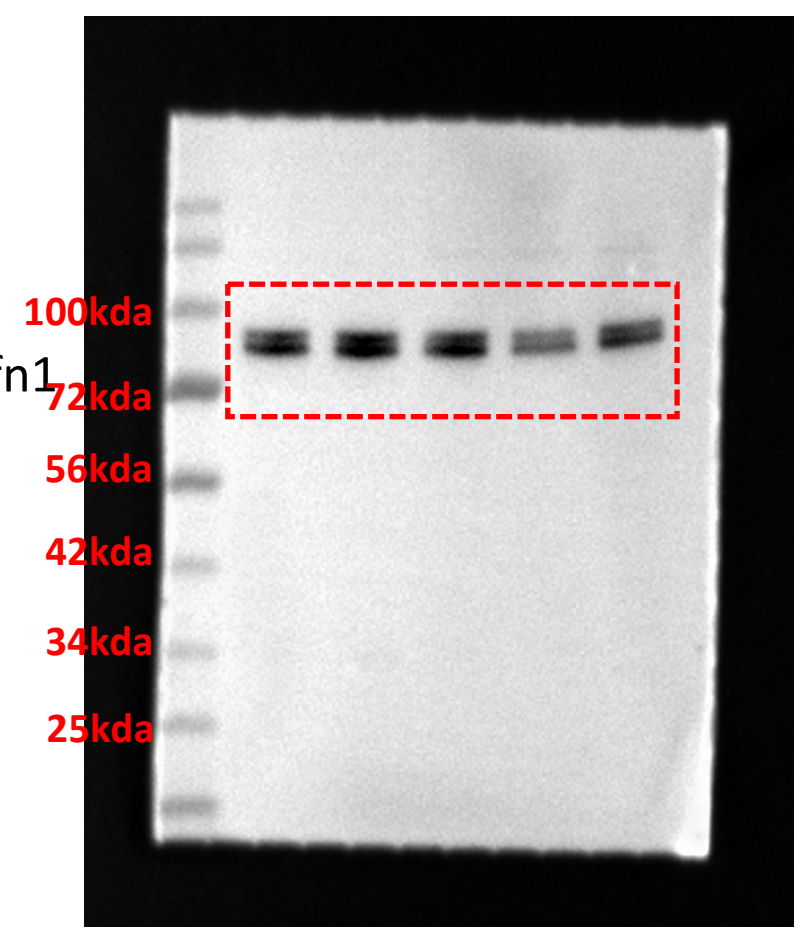

β-actin

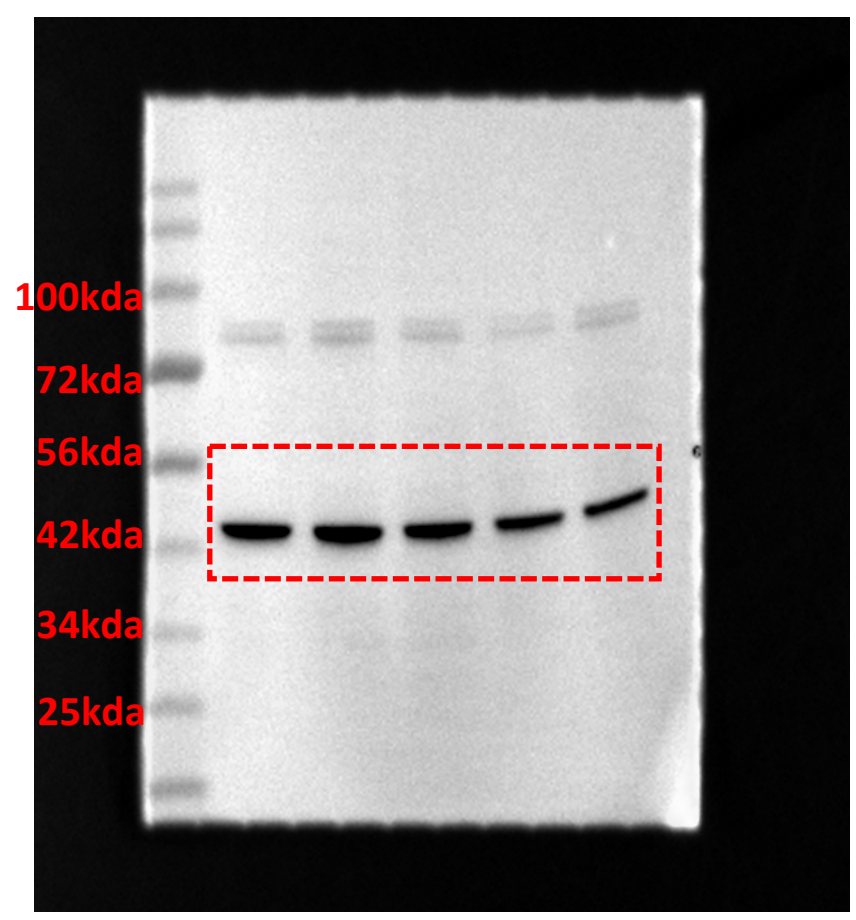

Figure 3n

DJ-1

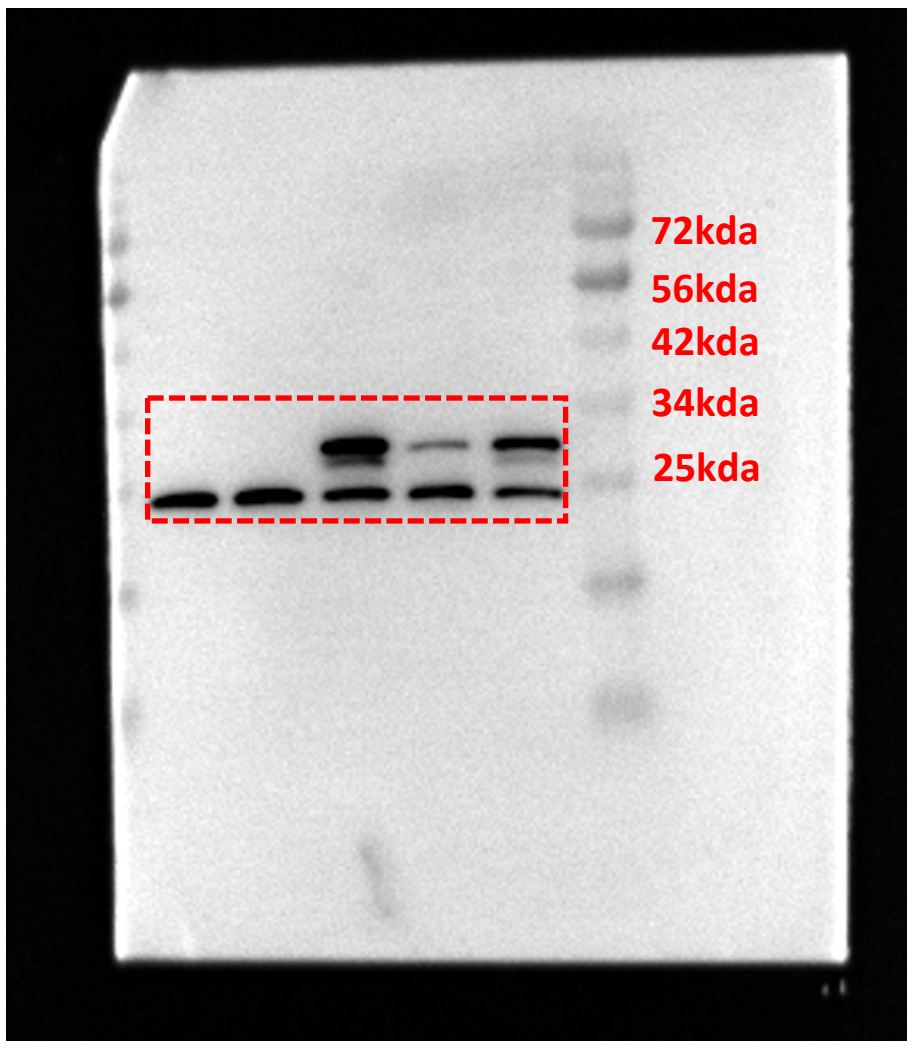

His

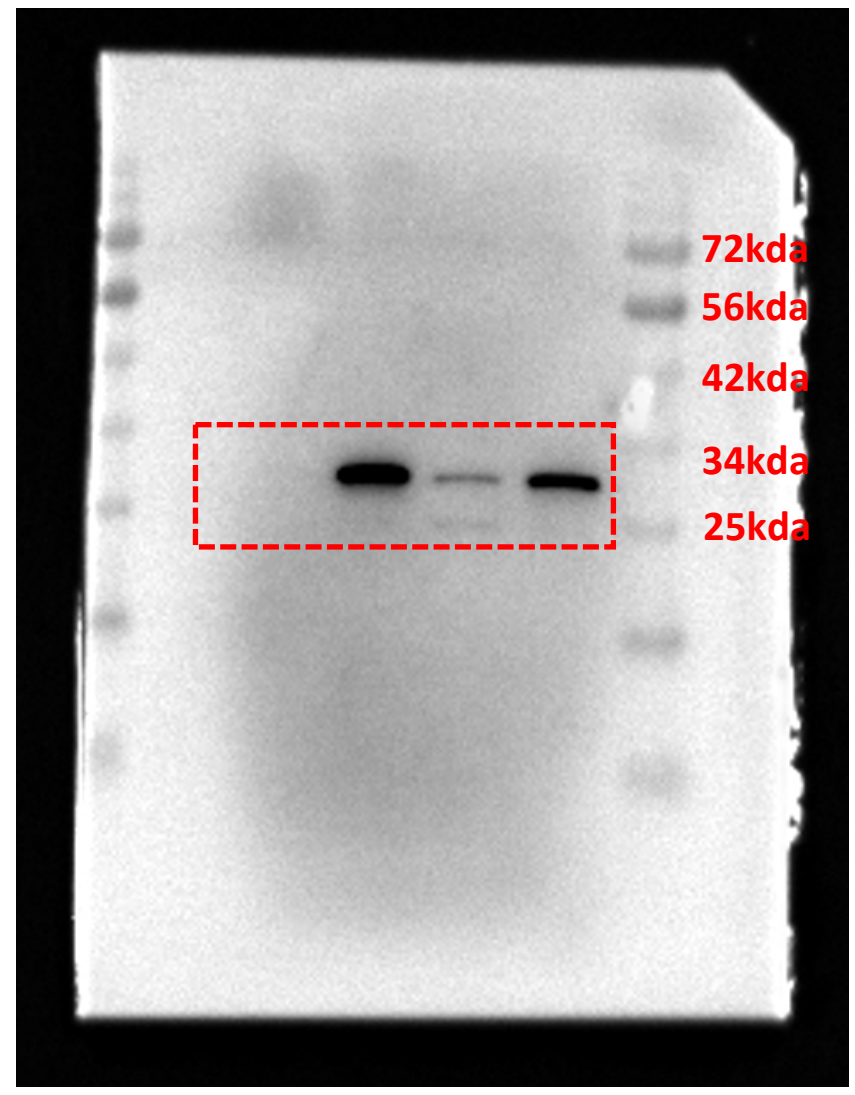

Myc

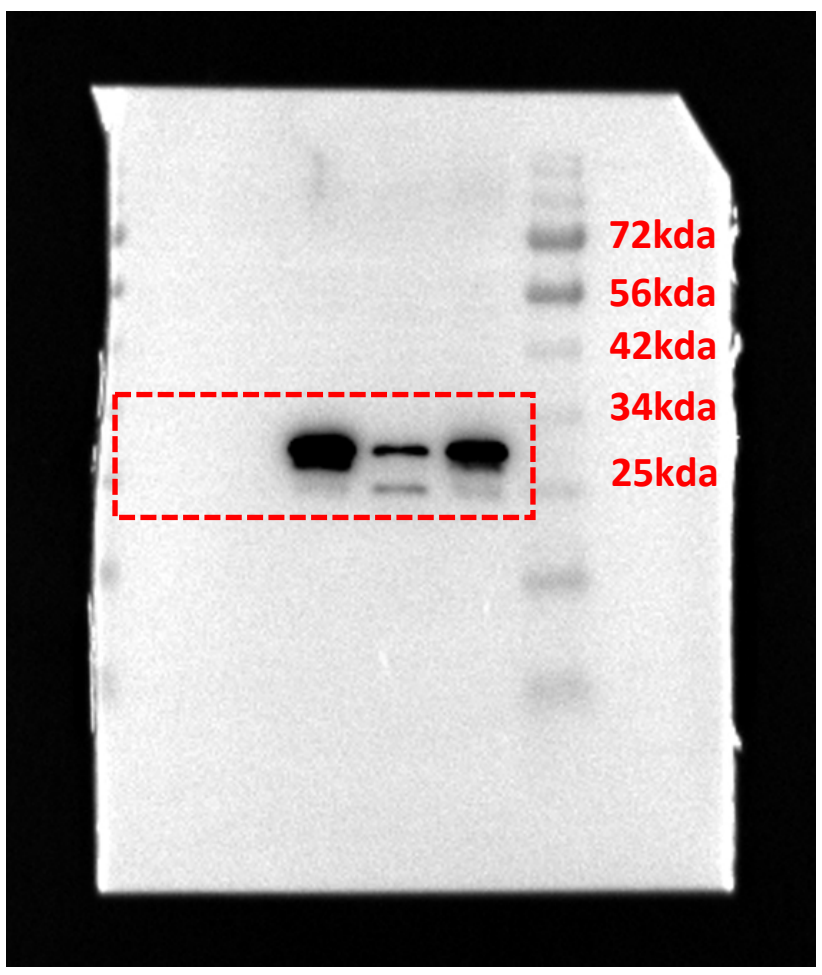

β-actin

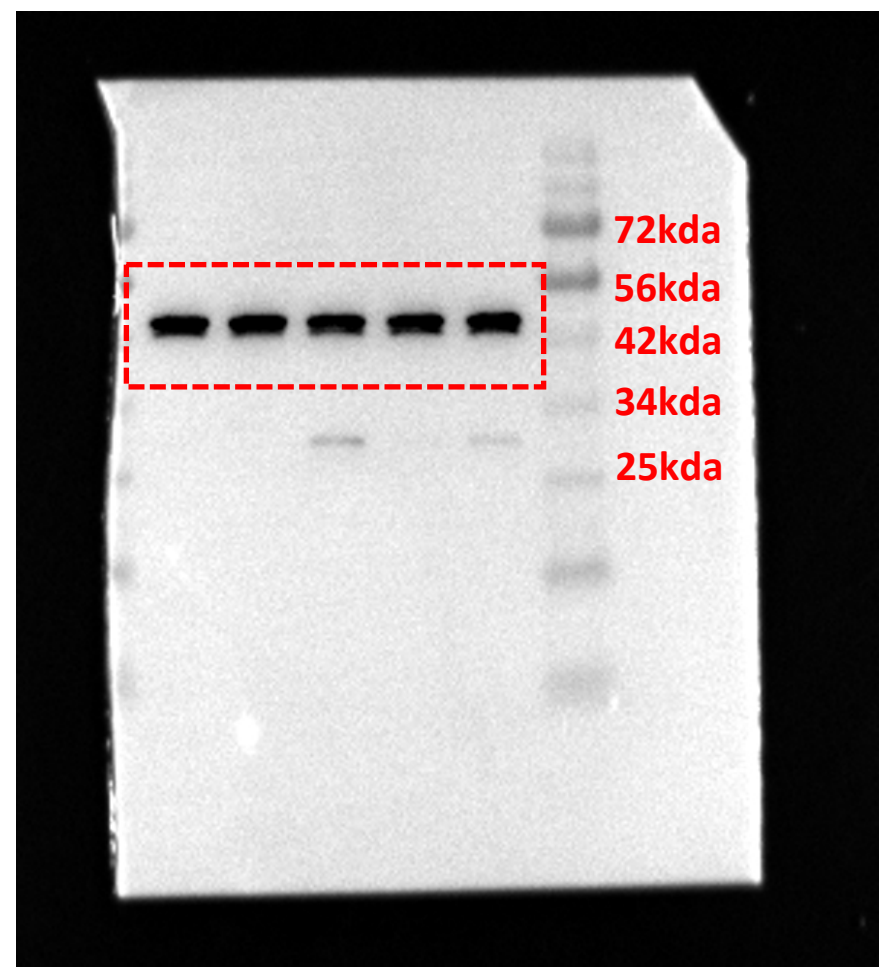

Figure 3s u

BNIP3

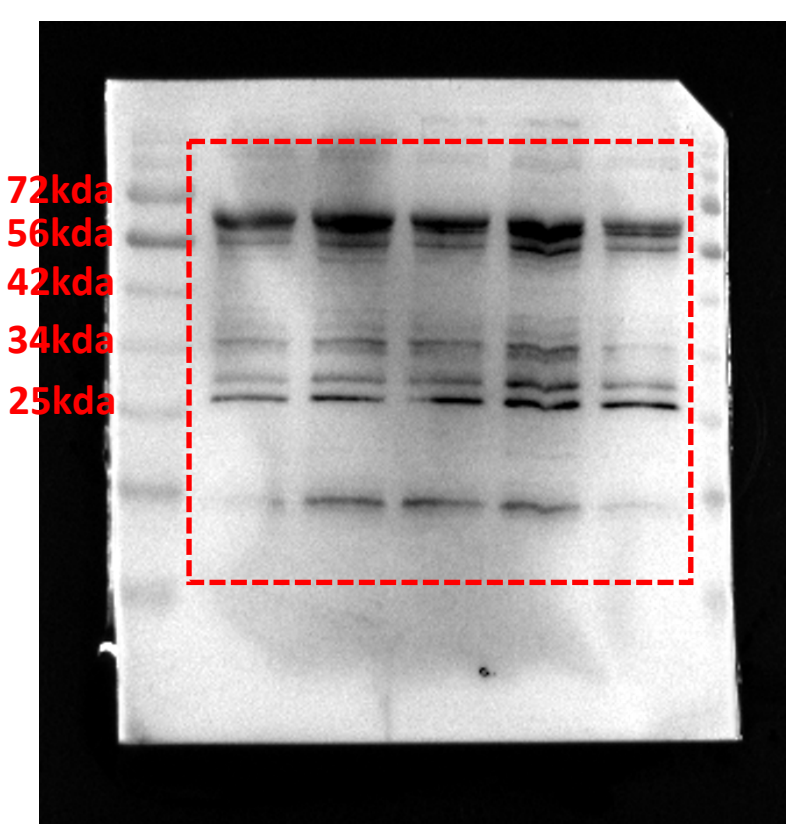

β-actin

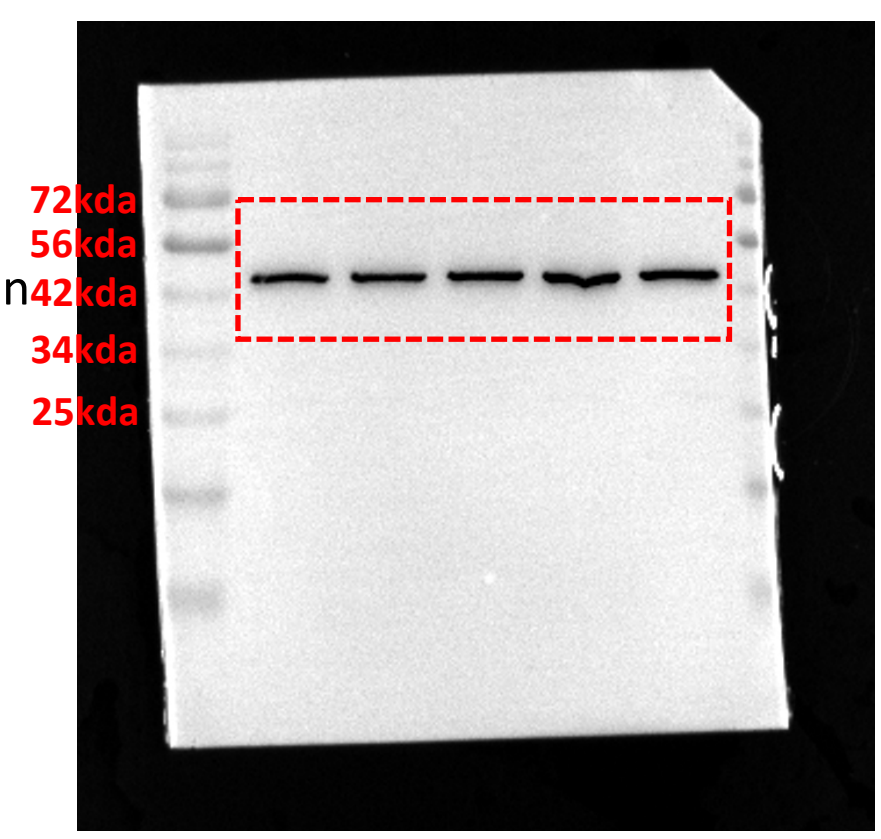

BNIP3L

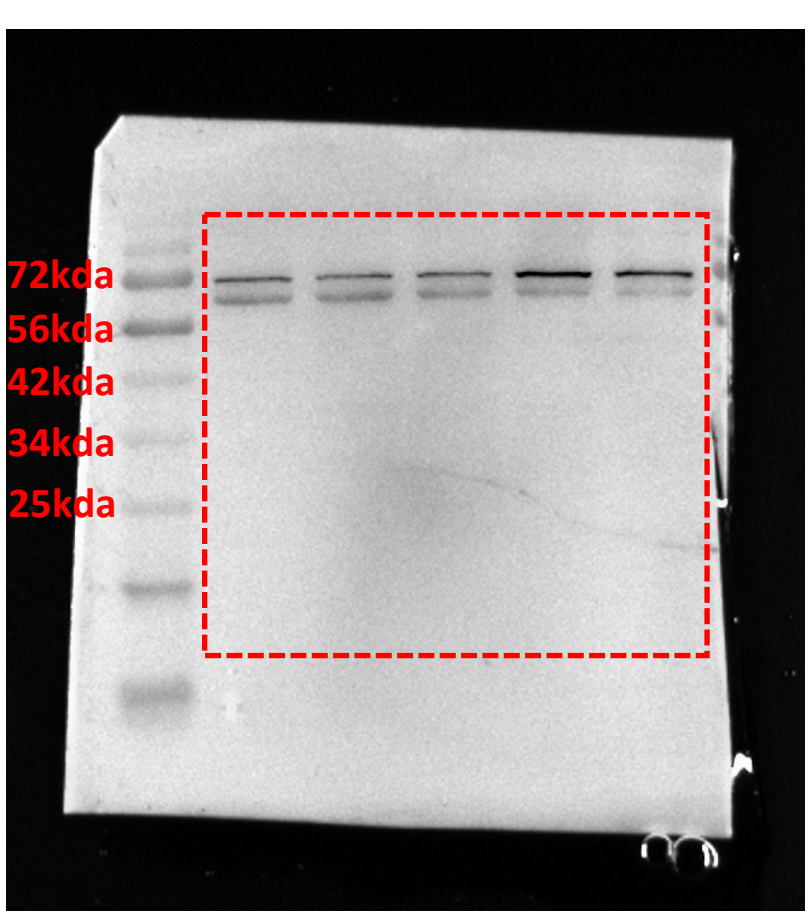

β-actin

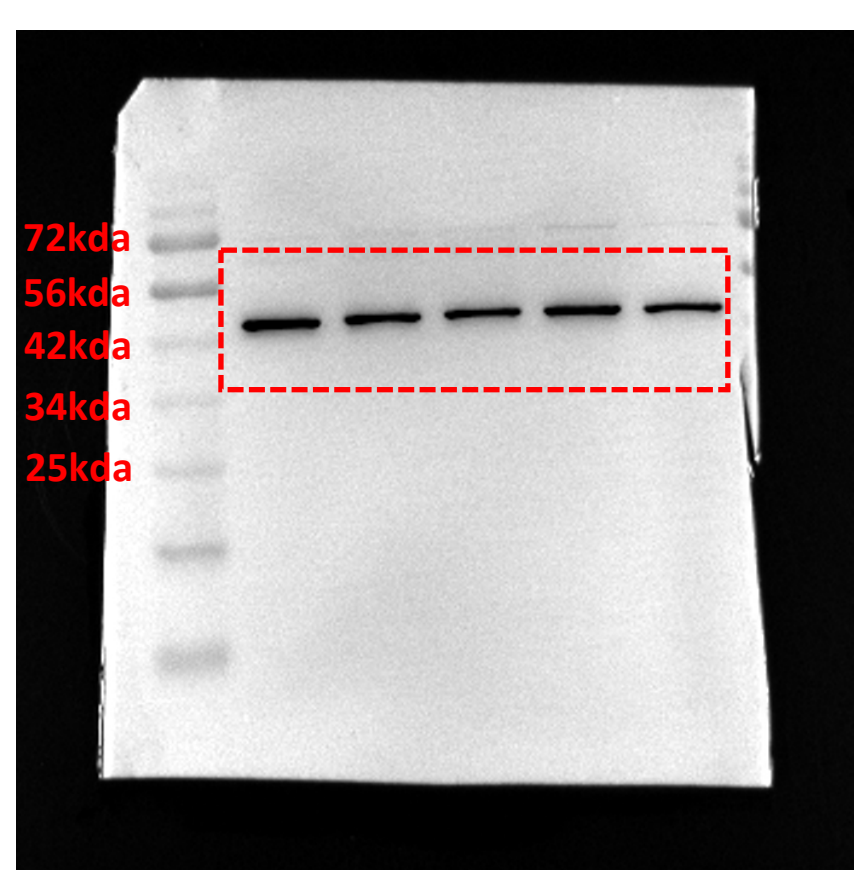

Figure 5a

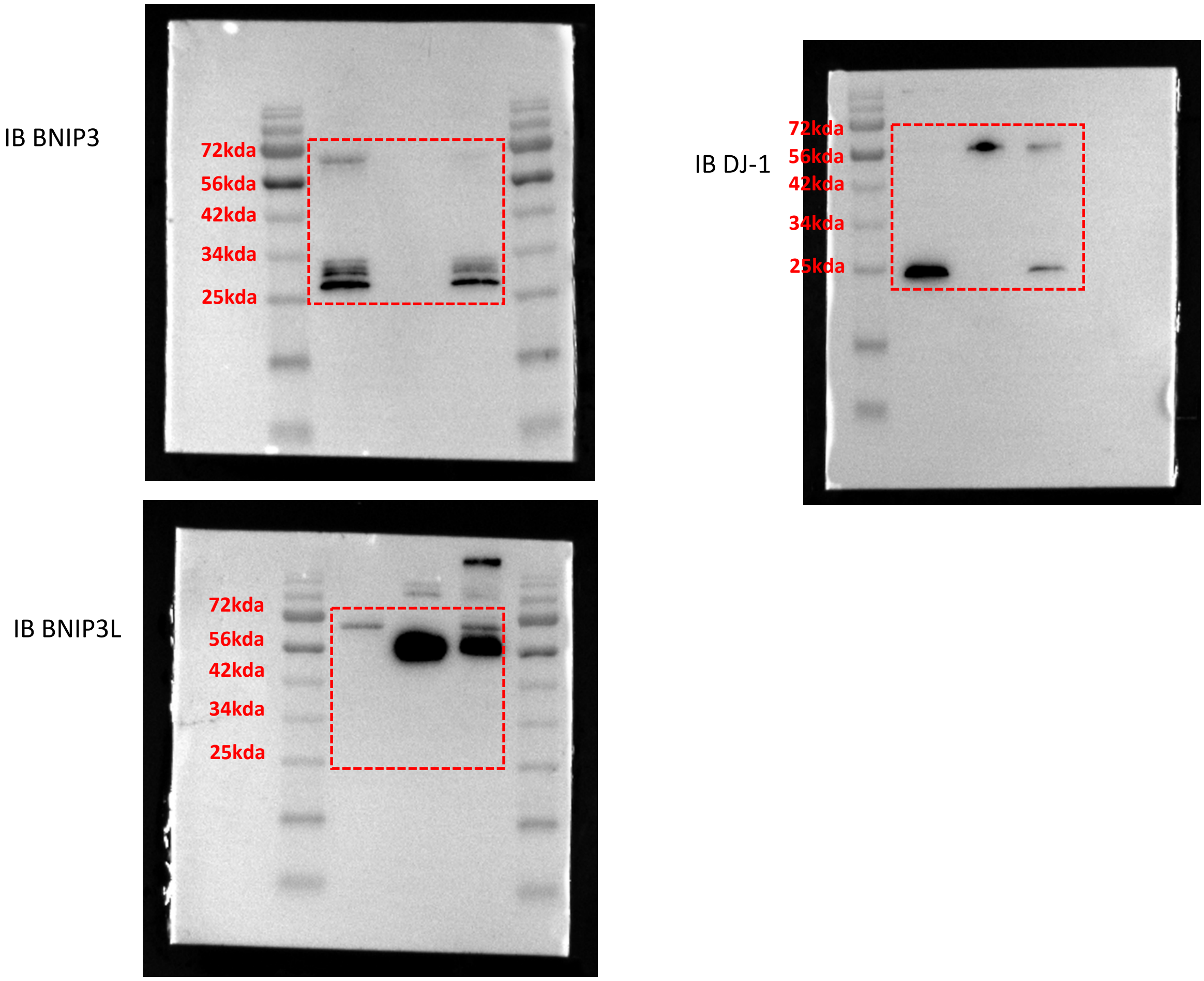

Figure 5b c

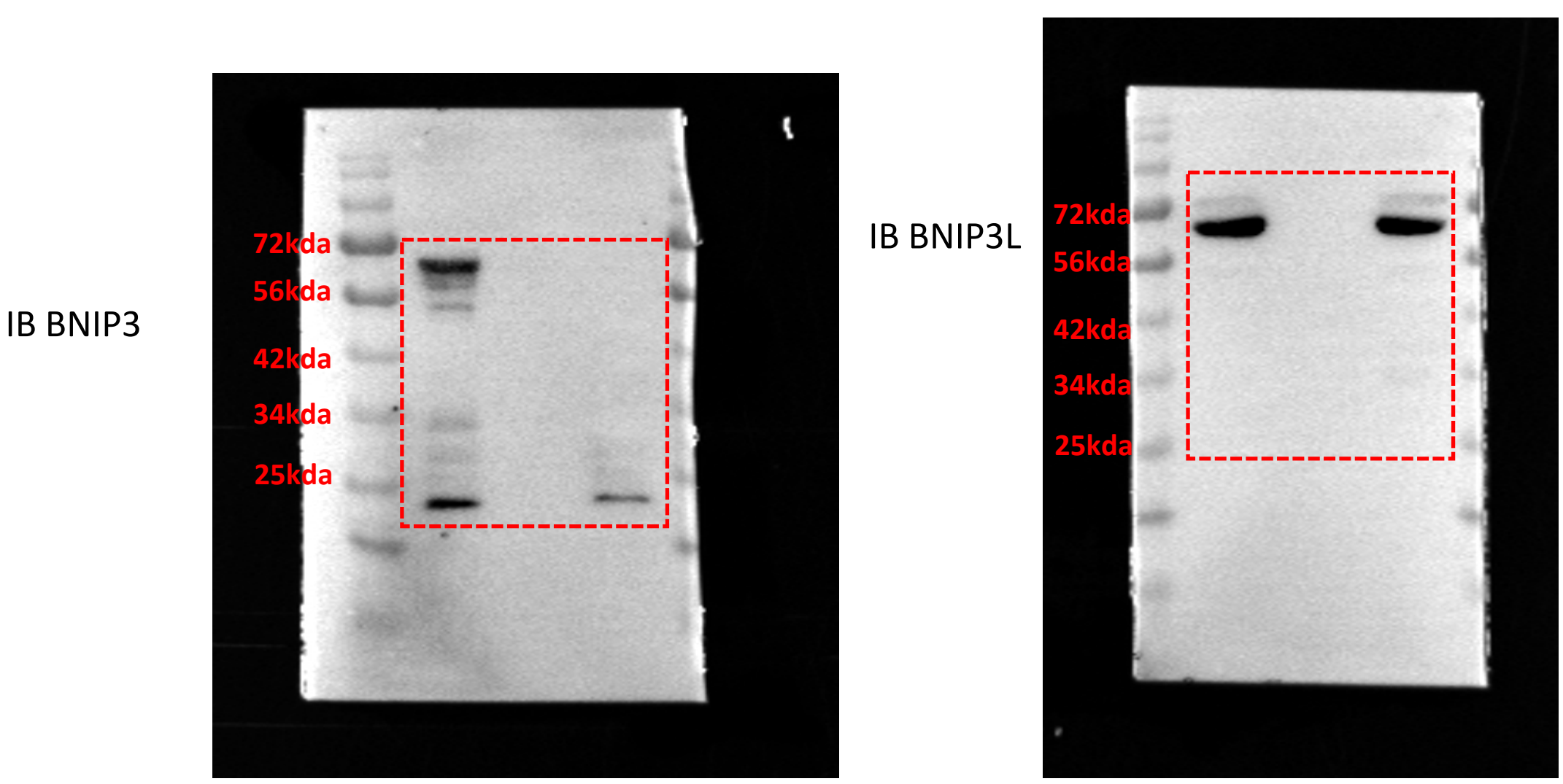

Figure 5d

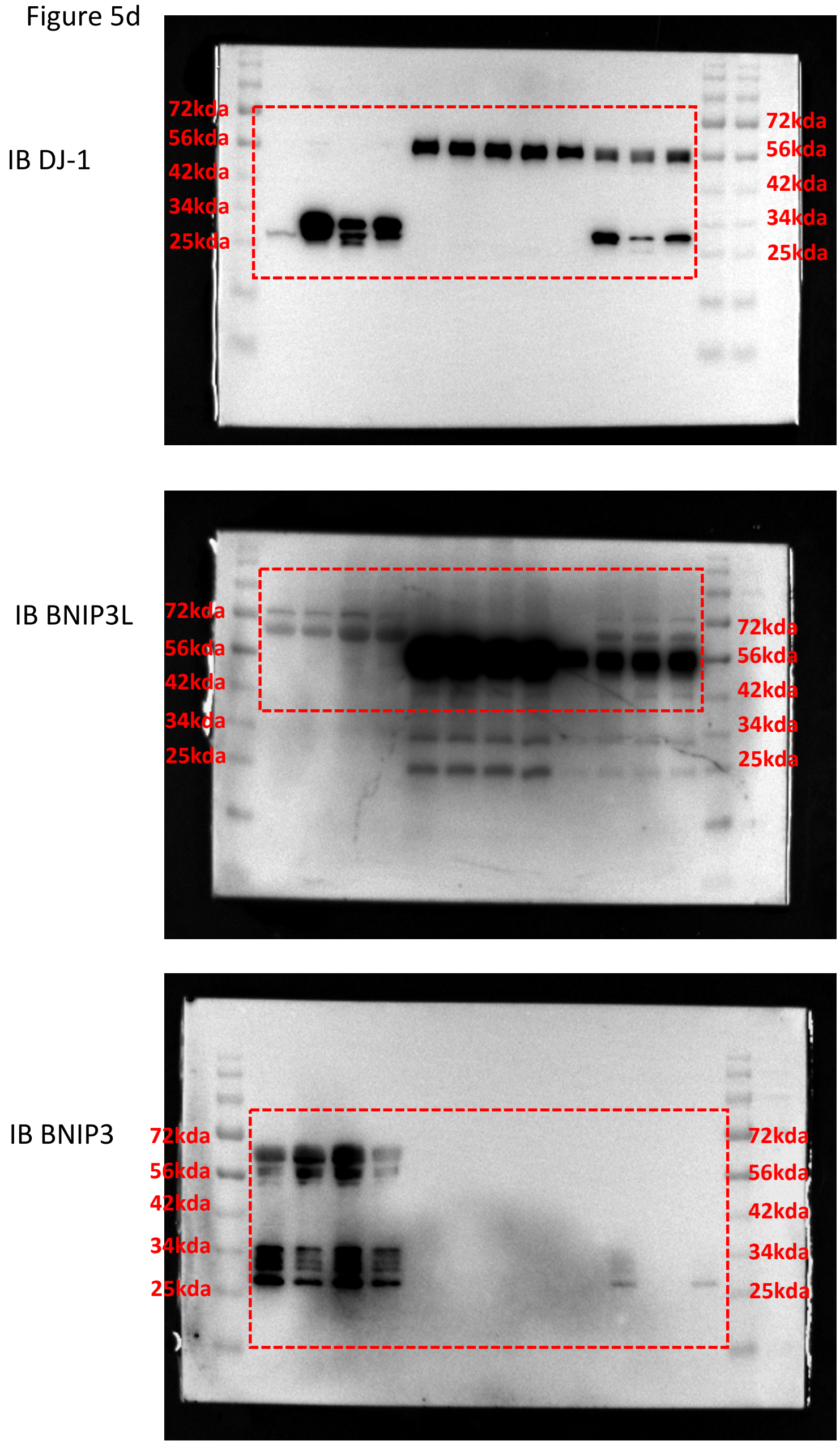

Figure 5e

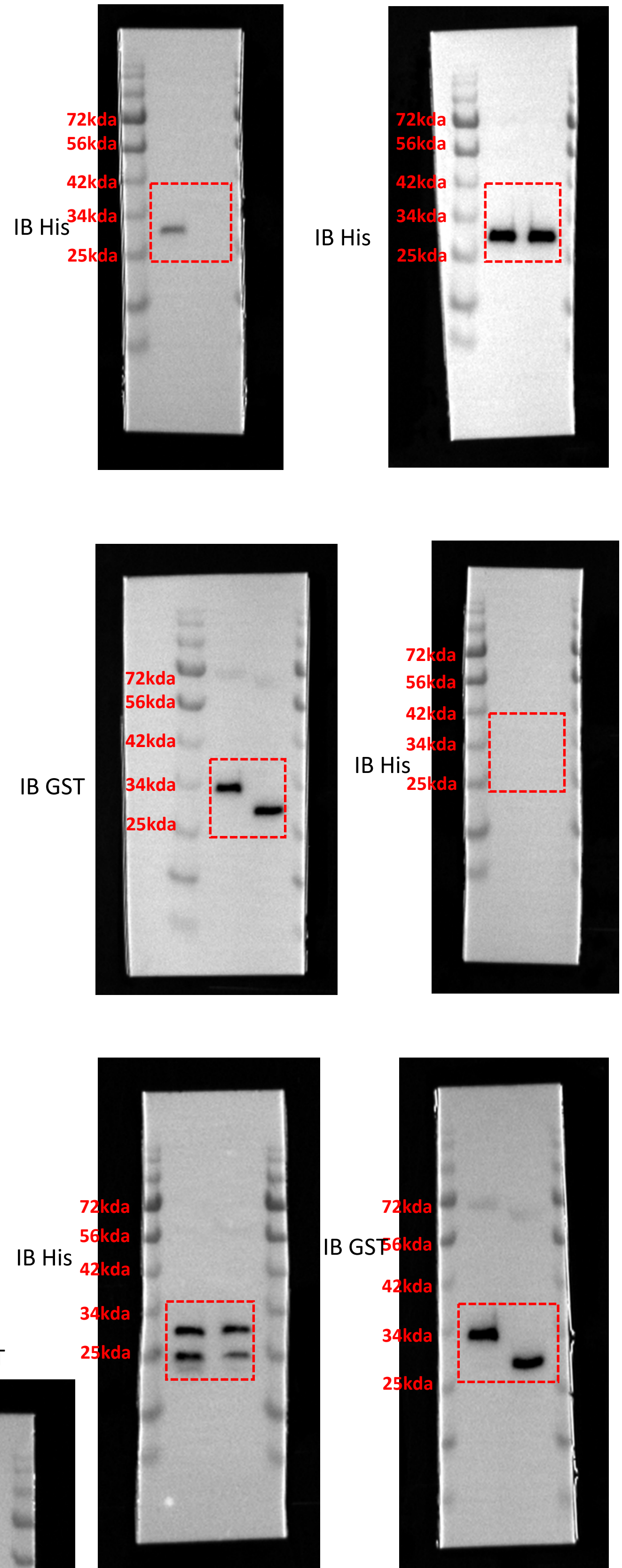

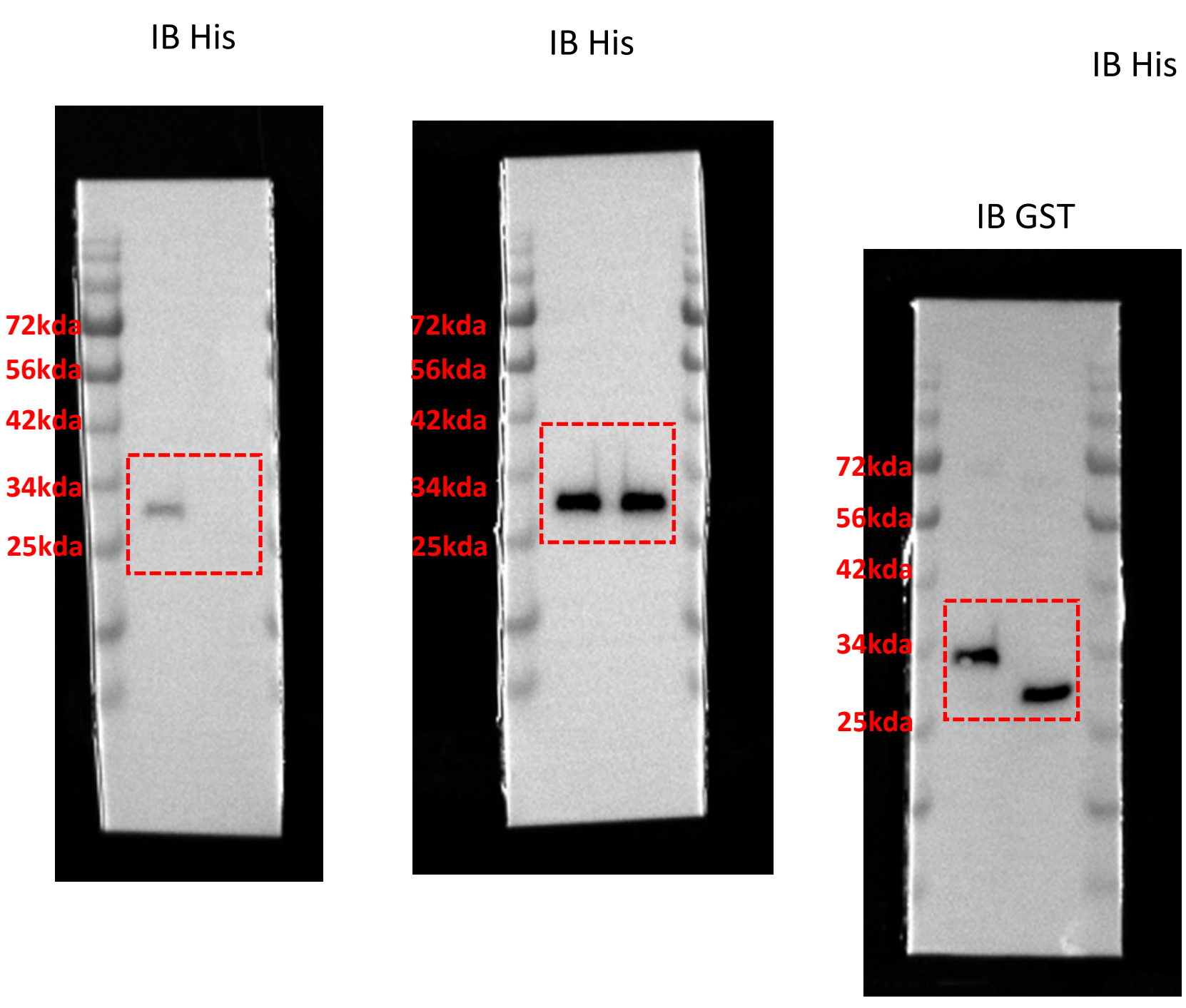

Figure 6a

GRP75

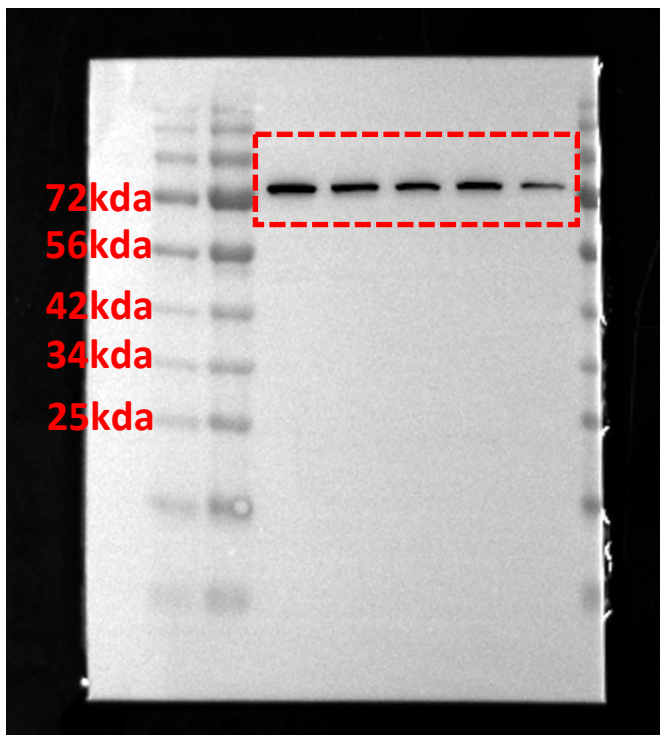

β-actin

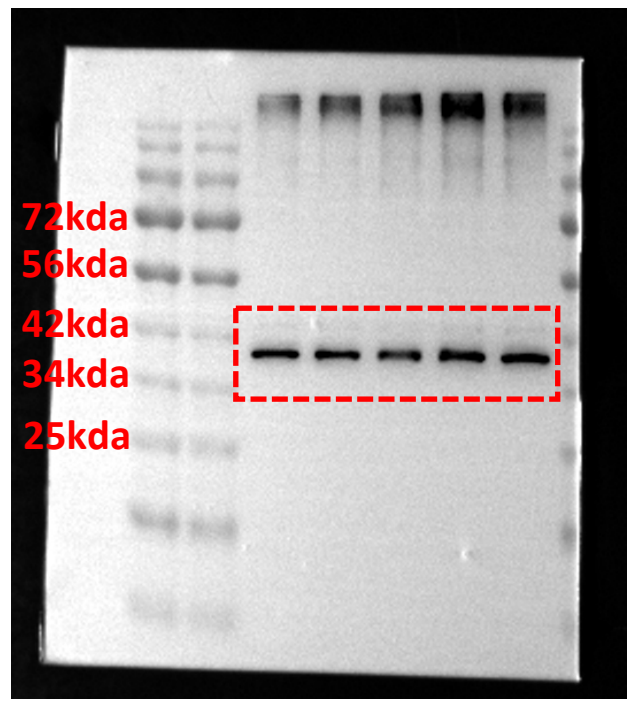

Figure 7e

VDAC1

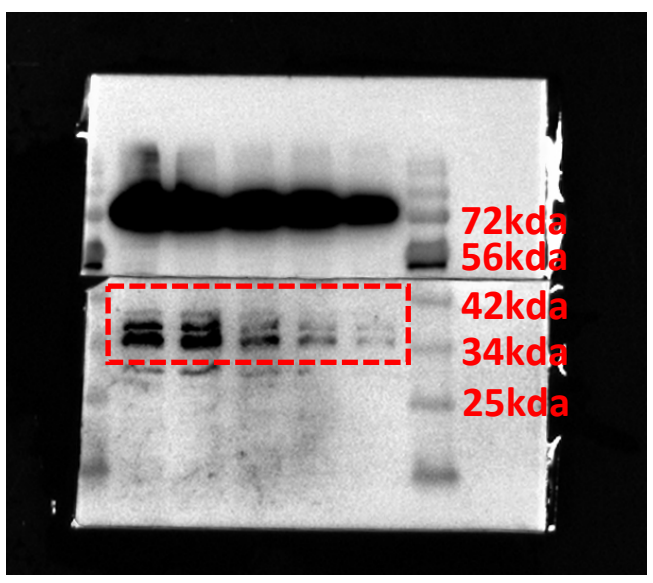

ERp72

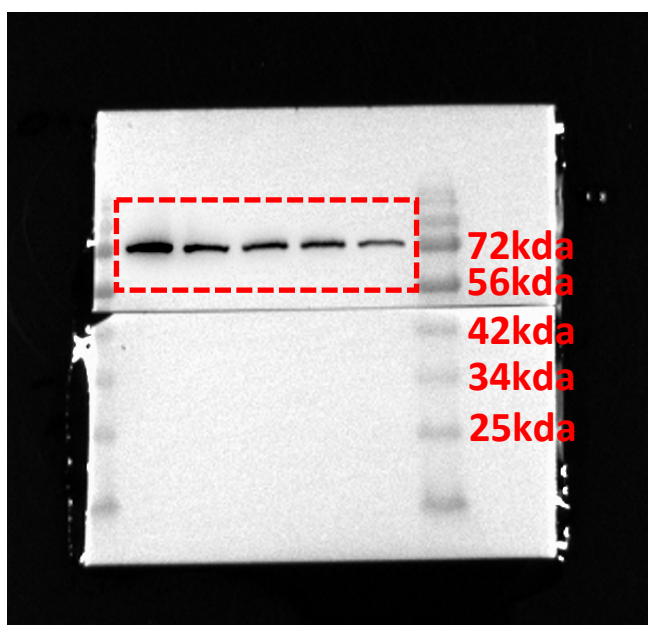

COX4

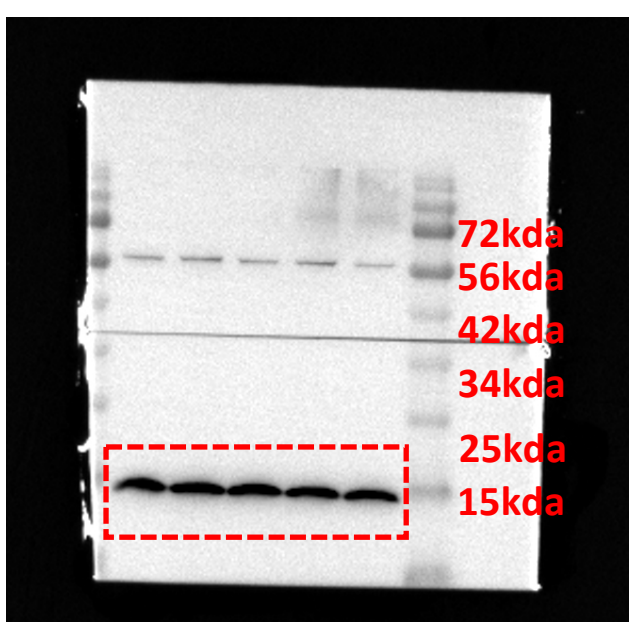

PDI

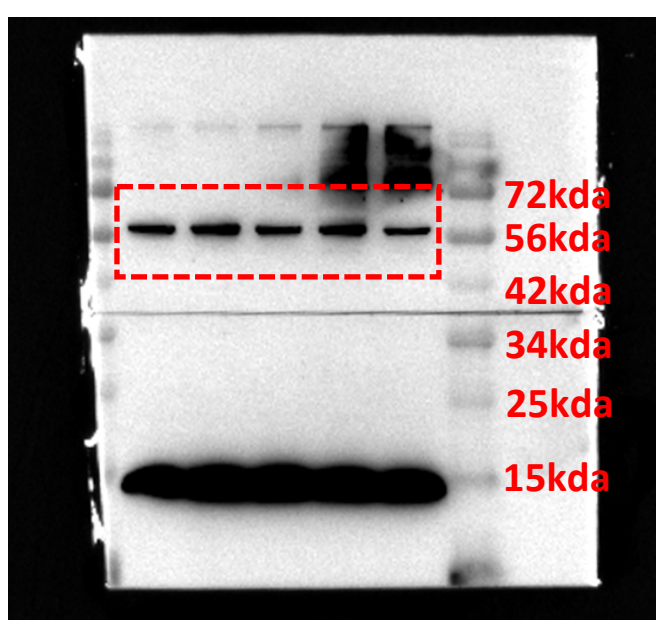

β-actin

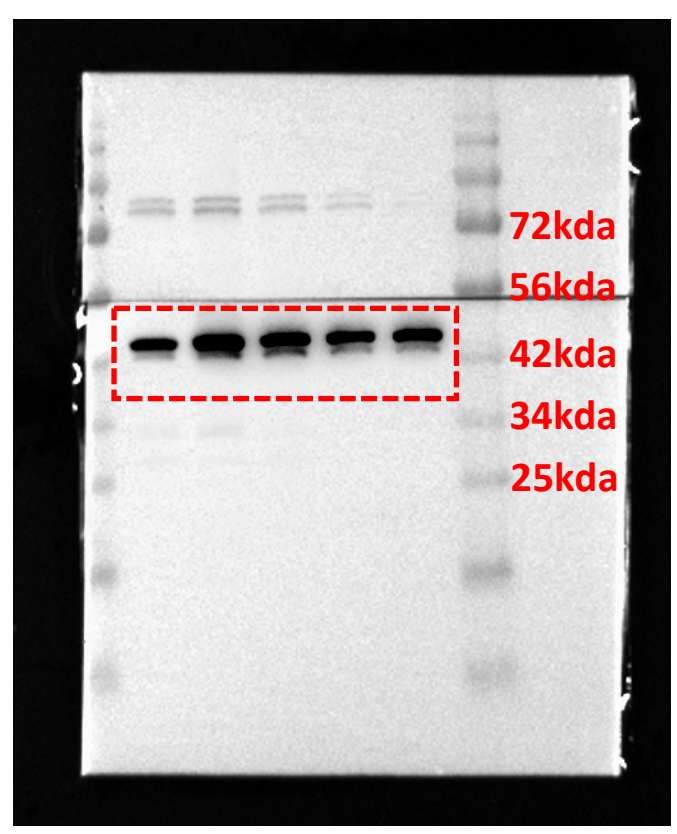

MFN1

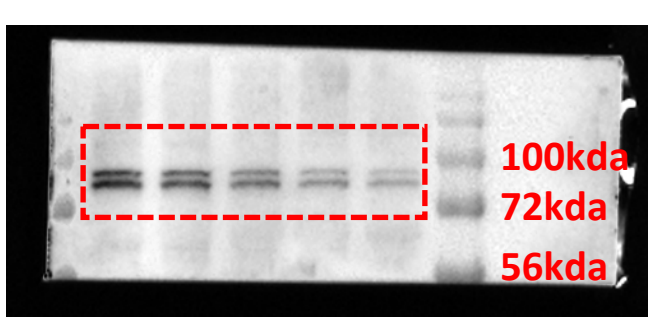

MFN2

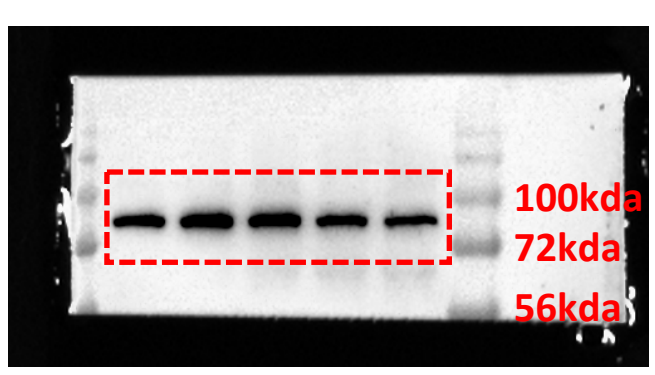

Supplementary Figure S1a c

DJ-1

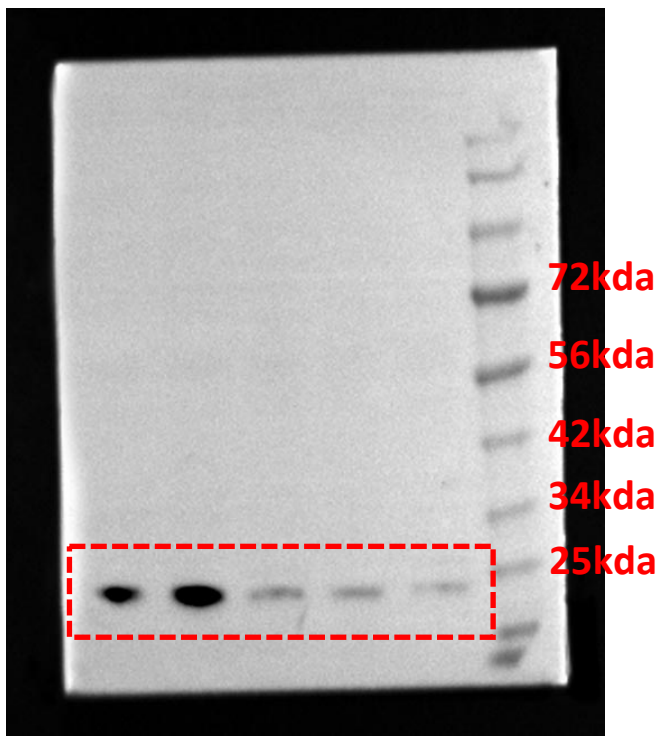

β-actin

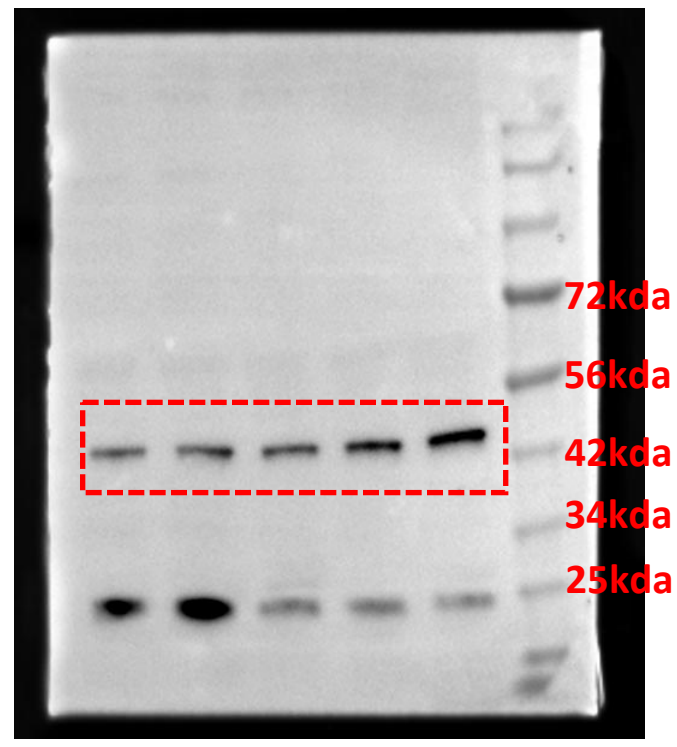

BNIP3

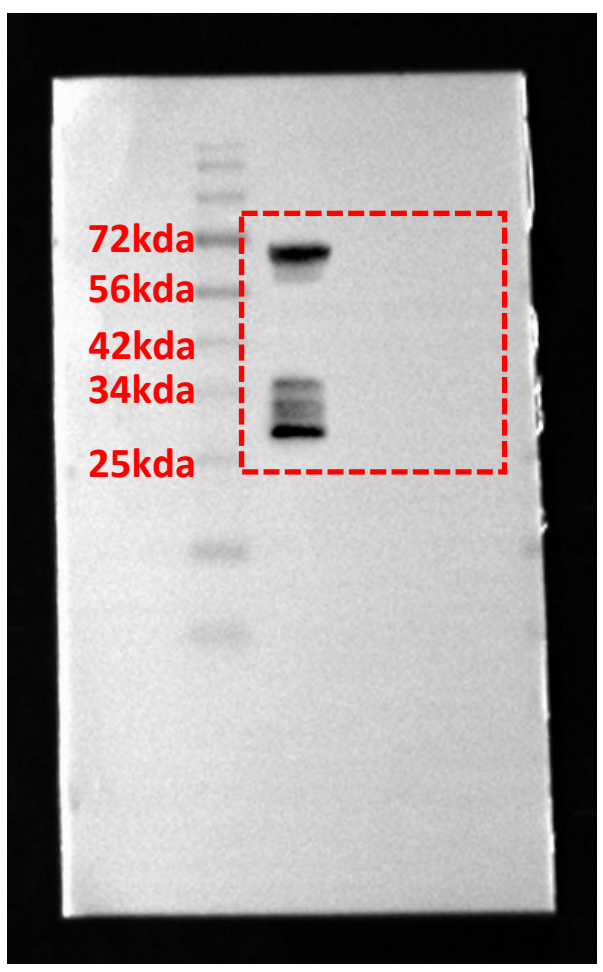

BNIP3L

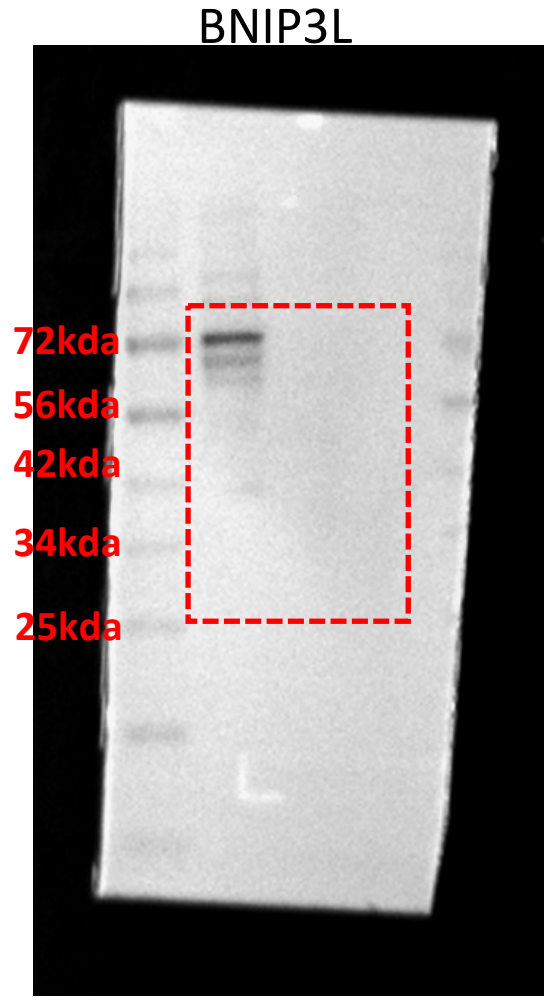

Supplementary Figure S2a c

BNIP3

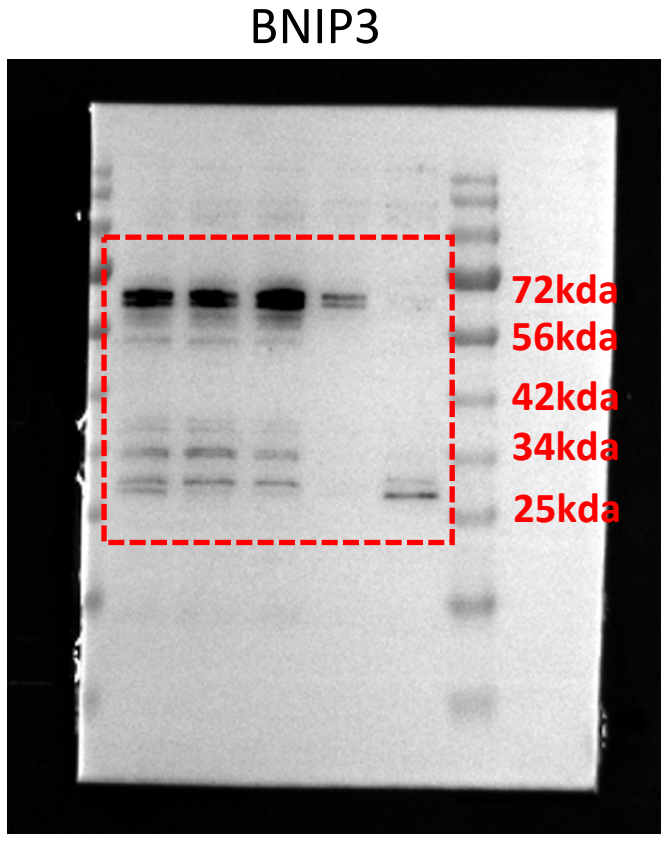

β-actin

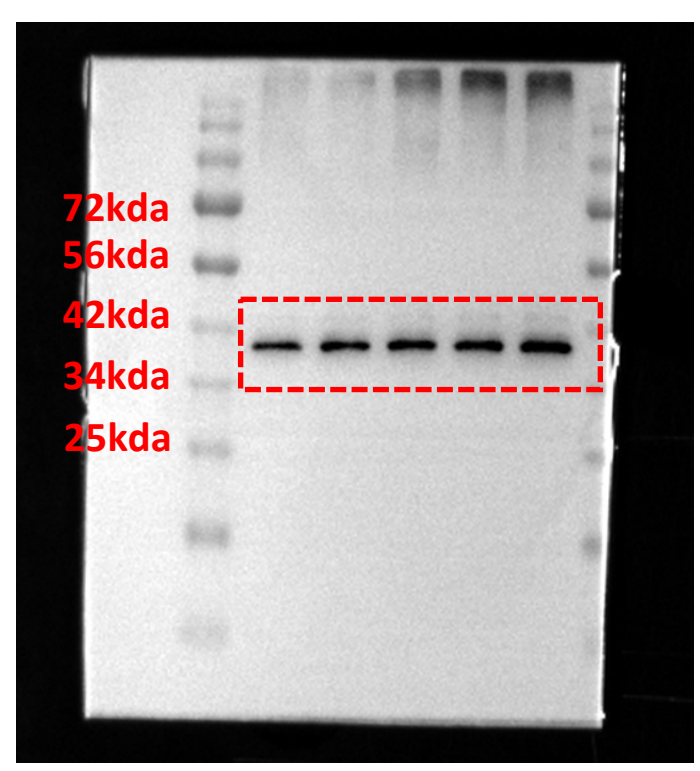

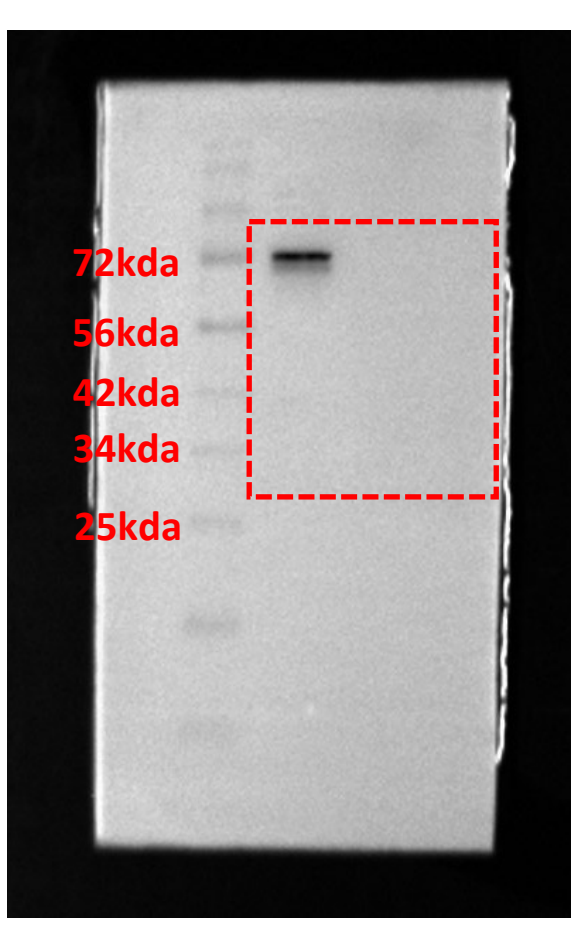

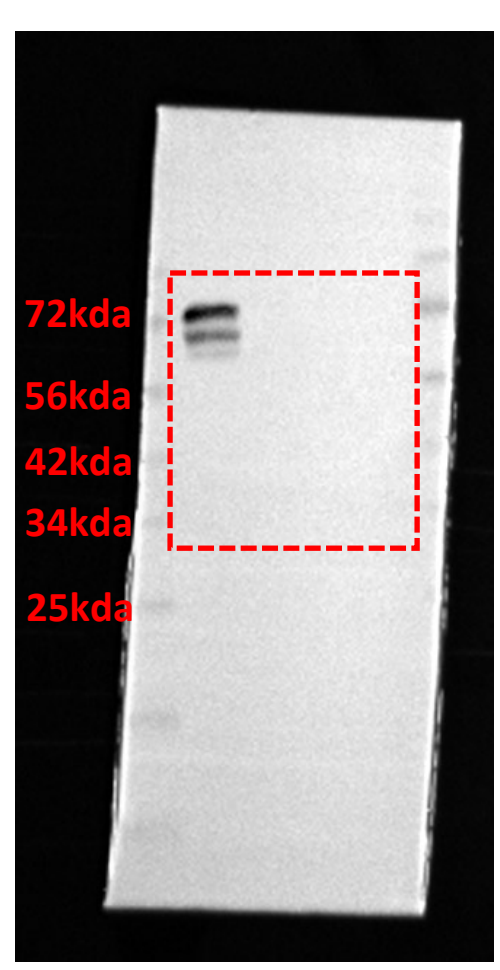

Supplementary Figure S3a c

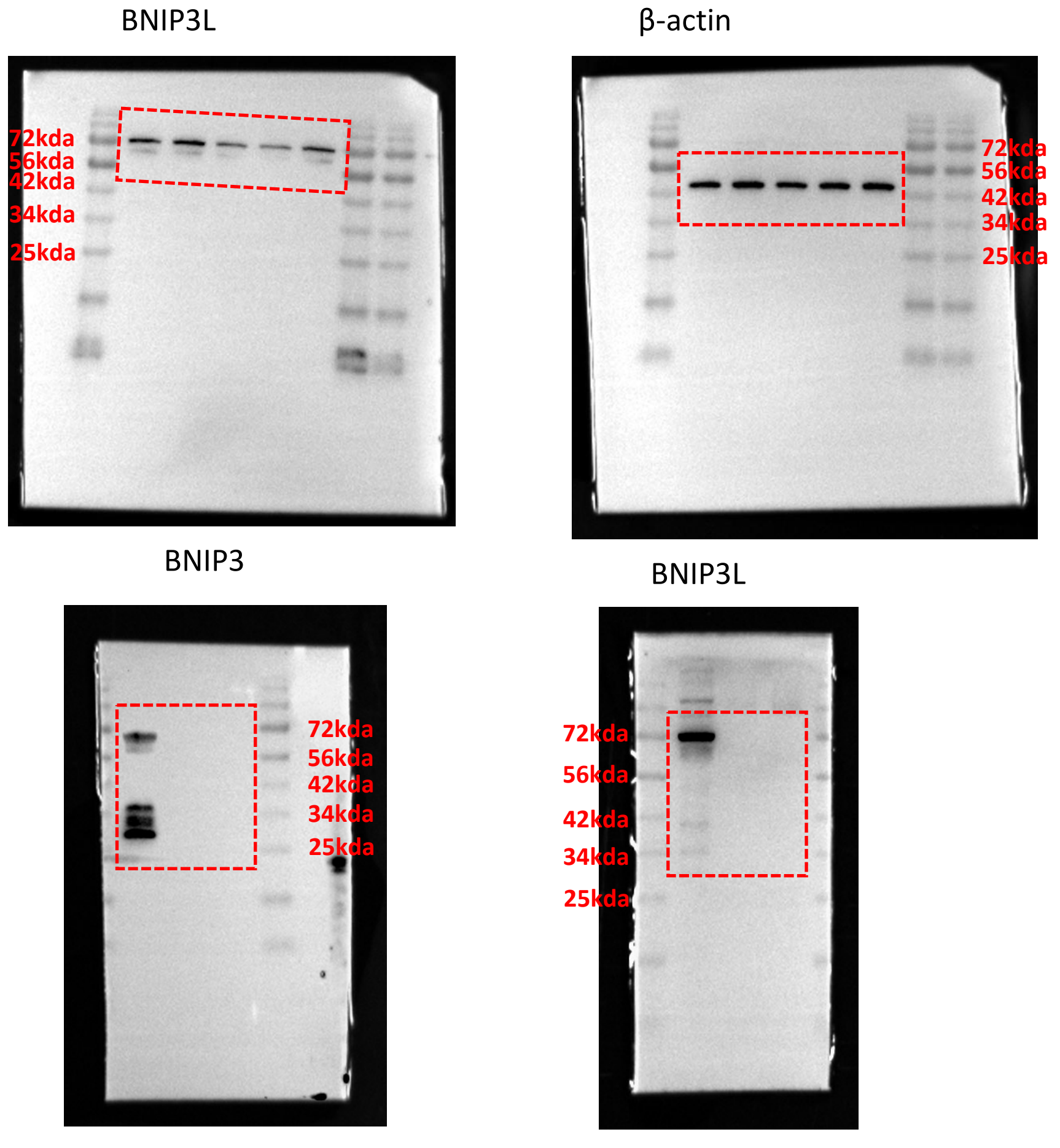

Supplementary Figure S4a c

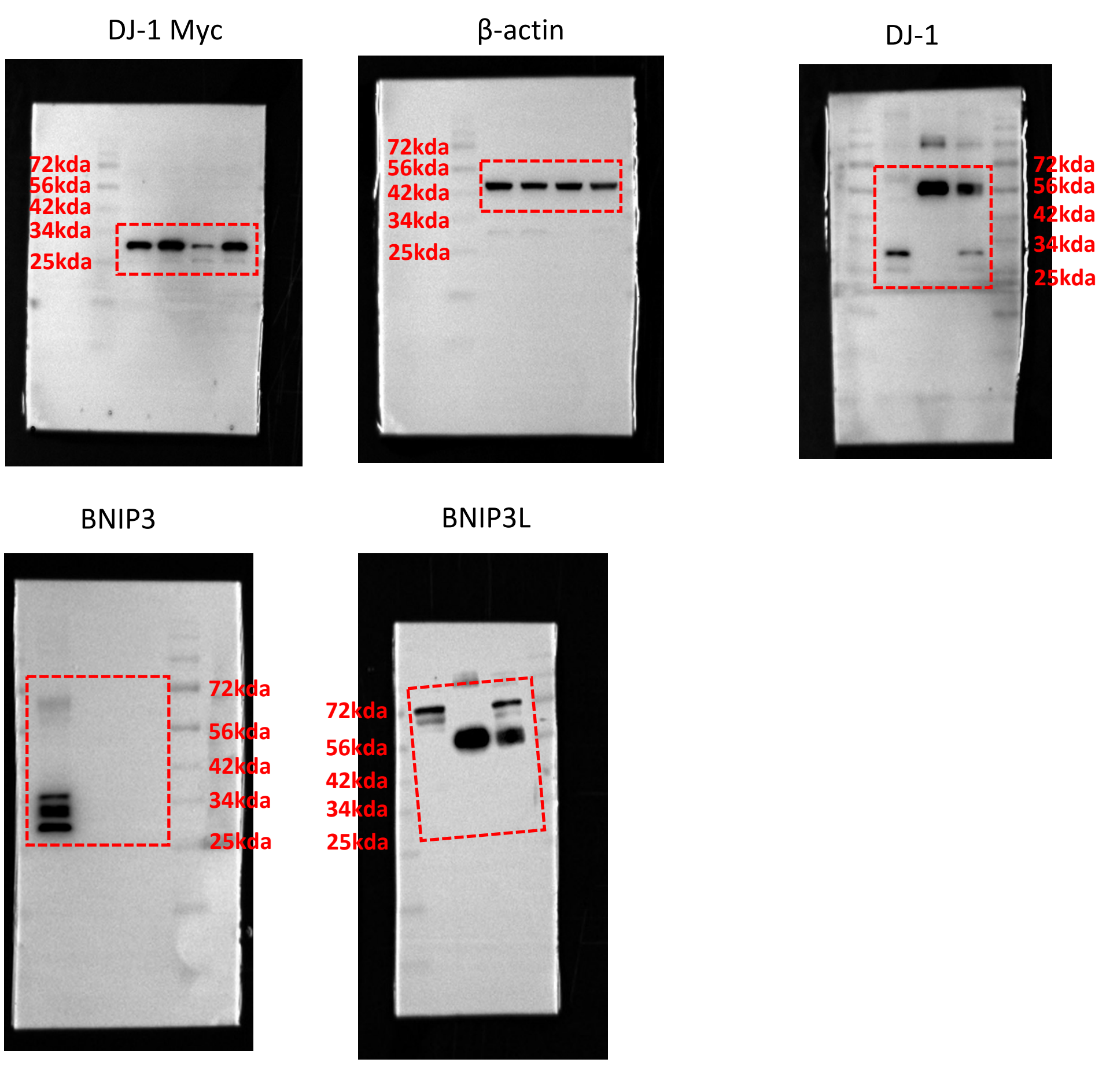

Supplementary Figure S5

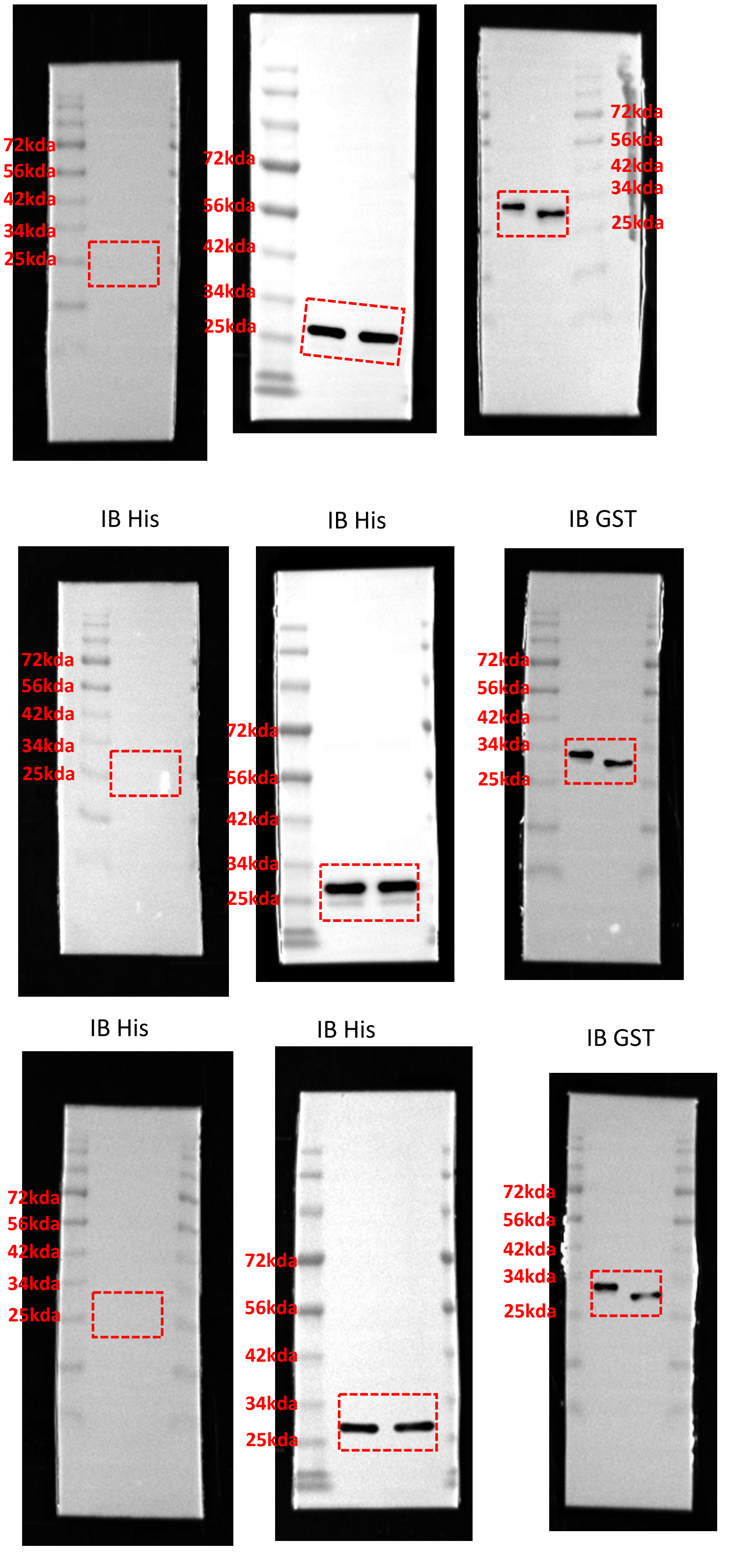

Supplement: Supplementary file 1 [file 13062_2026_928_MOESM2_ESM.pdf]
