# Supplementary material for: Effects of homozygous PARK7 gene mutations L166P and M26I on BNIP3/BNIP3L interactions and ER-mitochondria proximity
Source: Biol Direct. 2026 Aug 5;21:175. doi: 10.1186/s13062-026-00928-8 (PMC13563861; doi:10.1186/s13062-026-00928-8)
Supplement: Supplementary file 2 [file 13062_2026_928_MOESM1_ESM.docx]

**Effects of homozygous PARK7 gene mutations L166P and M26I on BNIP3/BNIP3L interactions and ER-mitochondria proximity**

**Supplementary Figures and Tables**

**Supplementary Figure S1: DJ-1 Knockdown Experiment**

DJ-1 knockdown efficiency and reciprocal BNIP3/BNIP3L co-immunoprecipitation.

**a, b** Representative Western blot and quantification showing DJ-1 levels in SH-SY5Y cells expressing siNC or siDJ-1. Data are shown as mean +/- SEM, N=3, **P<0.05, **P<0.01, ***P<0.001; ns, not significant* by one-way ANOVA with Tukey's post-hoc.

**c** Reciprocal co-immunoprecipitation of BNIP3 and BNIP3L in siDJ-1 cells. Cell lysates were immunoprecipitated with anti-BNIP3 or anti-BNIP3L and immunoblotted for the reciprocal partner. Input represents 20% of total lysate.

**Supplementary Figure S2: BNIP3 Knockdown Experiment**

BNIP3 knockdown efficiency and reciprocal BNIP3/BNIP3L co-immunoprecipitation.

**a, b** Representative Western blot and quantification showing BNIP3 levels in SH-SY5Y cells expressing siNC or siBNIP3. Data are shown as mean +/- SEM, N=3, ***P<0.01, ***P<0.001; ns, not significant* by one-way ANOVA with Tukey's post-hoc.

**c** Reciprocal co-immunoprecipitation of BNIP3 and BNIP3L in siBNIP3 cells. Cell lysates were immunoprecipitated with anti-BNIP3L or anti-BNIP3 and immunoblotted for the reciprocal partner. Input represents 20% of total lysate.

**Supplementary Figure S3: BNIP3L Knockdown Experiment**

BNIP3L knockdown efficiency and reciprocal BNIP3/BNIP3L co-immunoprecipitation.

**a, b** Representative Western blot and quantification showing BNIP3L levels in SH-SY5Y cells expressing siNC or siBNIP3L. Data are shown as mean +/- SEM, N=3, **P<0.05, **P<0.01, ns, not significant* by one-way ANOVA with Tukey's post-hoc.

**c** Reciprocal co-immunoprecipitation of BNIP3 and BNIP3L in siBNIP3L cells. Cell lysates were immunoprecipitated with anti-BNIP3 or anti-BNIP3L and immunoblotted for the reciprocal partner. Input represents 20% of total lysate.

**Supplementary Figure S4: MG132 Experiment**

MG132 partially restores DJ-1 L166P abundance but not detectable BNIP3 association.

**a, b** Representative Western blot and quantification of DJ-1 protein levels in SH-SY5Y cells expressing DJ-1 WT, L166P treated with 20μM MG132 for 8 h. Data are shown as mean +/- SEM, N=3, ***P<0.01, ***P<0.001* by one-way ANOVA with Tukey's post-hoc.

**c** Co-immunoprecipitation of BNIP3 with Myc-tagged DJ-1. Cell lysates were immunoprecipitated with rabbit anti-Myc antibody and immunoblotted for BNIP3 DJ-1 and BNIP3L. Input represents 20% of total lysate.

**Supplementary Figure S5: Mutant Peptide GST Pull-down**

GST pull-down analysis of a W2A/N8A mutant BNIP3 derived peptide. GST pull-down assay using GST-fused mutant peptide (DASSRPEAIPPKEF) incubated with lysates from SH-SY5Y cells expressing His-tagged DJ-1 WT, L166P, or M26I. Bound proteins were detected by anti-His and anti-GST Western blotting.

**Supplementary Figure S6**

**a** Delta Fmax quantification from the Fluo-4 RFU value. Delta Fmax was calculated as Peak RFU minus basal RFU.

**b** Delta AUC quantification from the Fluo-4 RFU value. Delta AUC was calculated as the summed RFU above basal RFU across scans 1-15.

**c** Time to peak. The time / the number of scans when the Fluo-4 signal reaches its peak.

**d** Peak response percentage. The percentage of each treatment group compared to the all-negative control group.

Data are shown as mean +/- SEM, N=3, ***P<0.01, ***P<0.001, ns, not significant* by one-way ANOVA with Tukey's post-hoc.

**Supplementary Figure S7**

Candidate residue contacts in AlphaFold3 generated DJ-1/BNIP3/BNIP3L three-protein models.

Summary of candidate residue contacts identified in AlphaFold3 generated models containing DJ-1 WT, L166P, or M26I together with BNIP3 and BNIP3L. The indicated distances and interaction types are computational predictions.

**Supplementary Table 1 Sequence of siRNA**

| **Names** | **Sequences 5’-3’** |
| --- | --- |
| **siDJ-1 #1 sense** | **CGACGAUCACUUAGAGAAACA** |
| **siDJ-1 #1 anti-sense** | **UUUCUCUAAGUGAUCGUCGCA** |
| **siDJ-1 #2 sense** | **UAUGUUAUAUGUUUACAAGCC** |
| **siDJ-1 #2 anti-sense** | **CUUGUAAACAUAUAACAUAAA** |
| **siDJ-1 #3 sense** | **UUUUAUGUUAUAUGUUUACAA** |
| **siDJ-1 #3 anti-sense** | **GUAAACAUAUAACAUAAAAAU** |
| **siBNIP3 #1 sense** | **GCCACGUCAUUGUGUUUAUU** |
| **siBNIP3 #1 anti-sense** | **AAUAAACACAAGUGACGUGGC** |
| **siBNIP3 #2 sense** | **AAAAGCAAGAAGAGUUUAGUG** |
| **siBNIP3 #2 anti-sense** | **CUAAACUCUUCUUGCUUUUGC** |
| **siBNIP3 #3 sense** | **AAGUAAUCCACUAACGAACCA** |
| **siBNIP3 #3 anti-sense** | **GUUCGUUAGUGGAUUACUUCU** |
| **siBNIP3L #1 sense** | **GAGAAUUGUUUCAGAGUUAUU** |
| **siBNIP3L #1 anti-sense** | **UAACUCUGAAACAAUUCUCUU** |
| **siBNIP3L #2 sense** | **AUUAAAAGACGCUAUUAGGAG** |
| **siBNIP3L #2 anti-sense** | **CCUAAUAGCGUCUUUUAAUUU** |
| **siBNIP3L #3 sense** | **GAUCAUGUUUGAUGUGGAAAU** |
| **siBNIP3L #3 anti-sense** | **UAACUGGUCAUCCUGAUGCUU** |

**Supplementary Table 2 Primary antibody of Western Blots**

| **Names** | **Brand and item number** | **Dilution for WB** |
| --- | --- | --- |
| **rabbit anti-PARK7/DJ-1** | **Proteintech, 11681-1-AP** | **1:5000** |
| **mouse anti-MFN1** | **Proteintech, 66776-1-Ig** | **1:10000** |
| **rabbit anti-Mitofusin-2 (D2D10)** | **Cell Signaling Technology, #9482** | **1:5000** |
| **mouse anti-BNIP3** | **Proteintech, 68091-1-Ig** | **1:20000** |
| **rabbit anti-BNIP3L** | **Proteintech, 12986-1-AP** | **1:5000** |
| **mouse anti-GFP tag** | **Proteintech, 66002-1-Ig** | **1:50000** |
| **mouse anti-6*His tag** | **Proteintech, 66005-1-Ig** | **1:50000** |
| **rabbit anti-MYC tag** | **Proteintech, 16286-1-AP** | **1:10000** |
| **mouse anti-MYC tag** | **Proteintech, 60003-2-Ig** | **1:10000** |
| **rabbit anti-GRP75** | **Proteintech, 14887-1-AP** | **1:10000** |
| **mouse anti-VDAC1** | **Proteintech, 66345-1-Ig** | **1:20000** |
| **rabbit anti-ERp72** | **Proteintech, 14712-1-AP** | **1:10000** |
| **rabbit anti-COXIV** | **Proteintech, 11242-1-AP** | **1:10000** |
| **mouse anti-PDI** | **Proteintech, 66422-1-Ig** | **1:10000** |
| **rabbit anti-GST tag** | **Proteintech, 10000-0-AP** | **1:10000** |
| **mouse anti-β-actin** | **Proteintech, 66009-1-Ig** | **1:20000** |
| **rabbit anti-β-actin** | **Proteintech, 20536-1-AP** | **1:20000** |
| **mouse anti-GAPDH** | **Proteintech, 60004-1-Ig** | **1:50000** |

**Supplementary Table 3 Primary antibody of Immunocytochemistry**

| **Names** | **Brand and item number** | **Dilution for ICC** |
| --- | --- | --- |
| **mouse anti-MFN1** | **Proteintech, 66776-1-Ig** | **1:1000** |
| **rabbit anti-Mitofusin-2** | **Cell Signaling Technology, #9482** | **1:200** |
| **mouse anti-BNIP3** | **Proteintech, 68091-1-Ig** | **1:500** |
| **rabbit anti-BNIP3L** | **Proteintech, 12986-1-AP** | **1:1000** |
| **mouse anti-His tag** | **Proteintech, 66005-1-Ig** | **1:500** |
| **rabbit anti-MYC tag** | **Proteintech, 16286-1-AP** | **1:500** |
| **mouse anti-VDAC1** | **Proteintech, 66345-1-Ig** | **1:500** |
| **rabbit anti-ERp72** | **Proteintech, 14712-1-AP** | **1:500** |
| **rabbit anti-COXIV** | **Proteintech, 11242-1-AP** | **1:1000** |
| **mouse anti-PDI** | **Proteintech, 66422-1-Ig** | **1:500** |
| **mouse anti-Calnexin** | **Proteintech, 66903-1-Ig** | **1:500** |
| **rabbit anti-GRP75** | **Proteintech, 14887-1-AP** | **1:500** |
